# Automated eukaryotic gene structure annotation using EVidenceModeler and the Program to Assemble Spliced Alignments

**DOI:** 10.1186/gb-2008-9-1-r7

**Published:** 2008-01-11

**Authors:** Brian J Haas, Steven L Salzberg, Wei Zhu, Mihaela Pertea, Jonathan E Allen, Joshua Orvis, Owen White, C Robin Buell, Jennifer R Wortman

**Affiliations:** 1 J Craig Venter Institute, The Institute for Genomic Research, Rockville, 9712 Medical Center Drive, Maryland 20850, USA; 2 Broad Institute of MIT and Harvard, 7 Cambridge Center, Cambridge, Massachusetts 02142, USA; 3 Center for Bioinformatics and Computational Biology, Department of Computer Science, 3125 Biomolecular Sciences Bldg #296, University of Maryland, College Park, Maryland 20742, USA; 4 Computation Directorate, Lawrence Livermore National Laboratory, 7000 East Avenue, Livermore, California 94550, USA; 5 Institute for Genome Sciences, University of Maryland Medical School, Baltimore, Maryland 21201, USA; 6 Department of Plant Biology, Michigan State University, East Lansing, Michigan 48824, USA

## Abstract

EVidenceModeler (EVM) is presented as an automated eukaryotic gene structure annotation tool that reports eukaryotic gene structures as a weighted consensus of all available evidence. EVM, when combined with the Program to Assemble Spliced Alignments (PASA), yields a comprehensive, configurable annotation system that predicts protein-coding genes and alternatively spliced isoforms. Our experiments on both rice and human genome sequences demonstrate that EVM produces automated gene structure annotation approaching the quality of manual curation.

## Abstract 2

EVidenceModeler (EVM) is an automated annotation tool that predicts protein-coding regions, alternatively spliced transcripts and untranslated regions of eukaryotic genes.

## Background

Accurate and comprehensive gene discovery in eukaryotic genome sequences requires multiple independent and complementary analysis methods including, at the very least, the application of *ab initio *gene prediction software and sequence alignment tools. The problem is technically challenging, and despite many years of research no single method has yet been able to solve it, although numerous tools have been developed to target specialized and diverse variations on the gene finding problem (for review [1,2]). Conventional gene finding software employs probabilistic techniques such as hidden Markov models (HMMs). These models are employed to find the most likely partitioning of a nucleotide sequence into introns, exons, and intergenic states according to a prior set of probabilities for the states in the model. Such gene finding programs, including GENSCAN [3], GlimmerHMM [4], Fgenesh [5], and GeneMark.hmm [6], are effective at identifying individual exons and regions that correspond to protein-coding genes, but nevertheless they are far from perfect at correctly predicting complete gene structures, differing from correct gene structures in exon content or position [7-10].

The correct gene structures, or individual components including introns and exons, are often apparent from spliced alignments of homologous transcript or protein sequences. Many software tools are available that perform these alignment tasks. Tools used to align expressed sequence tags (ESTs) and full-length cDNAs (FL-cDNAs) to genomic sequence include EST_GENOME [11], AAT [12], sim4 [13], geneseqer [14], BLAT [15], and GMAP [16], among numerous others. The list of programs that perform spliced alignments of protein sequences to DNA are much fewer, including the multifunctional AAT, exonerate [17], and PMAP (derived from GMAP). An extension of spliced protein alignment that includes a probabilistic model of eukaryotic gene structure is implemented in GeneWise [18], a popular homology-based gene predictor that serves a critical role in the Ensembl automated genome annotation pipeline [19]. In most cases, the spliced protein alignments and transcript alignments (derived from ESTs) provide evidence for only part of the gene structure, delineating introns, complete internal exons, and potential portions of other exons at their alignment termini.

A comprehensive approach to eukaryotic gene structure annotation should utilize both the information intrinsic to the genome sequence itself, as is done by *ab initio *gene prediction software, and any extrinsic data in the form of homologies to other known sequences, including proteins, transcripts, or conserved regions revealed from cross-genome comparisons. Some of the most recent *ab initio *gene finding software is able to utilize such extrinsic data to improve upon gene finding accuracy. Examples of such software are numerous, and each falls within a certain niche based on the form of extrinsic data utilized. TWINSCAN [20], for example, uses an 'informant' genome to condition the probabilities of exons and introns in a closely related genome. Subsequently, TWINSCAN_EST [21] combined spliced transcript alignments with the intrinsic data, and finally N-SCAN [22] (also known as TWINSCAN 3.0) and N-SCAN_EST [21] utilized cross-genome homologies to multiple related genome sequences in the context of a phylogenetic framework. Other tools, including Augustus [23], Genie [24], and ExonHunter [25] include mechanisms to incorporate extrinsic data into the *ab initio *gene prediction framework to improve accuracy further. Each of these programs analyzes and predicts genes along a single target genome sequence, while using homologies detected to other sequences. A more specialized approach to gene-finding is employed by the tools SLAM [26] and TWAIN [27], which consider homologies between two related genome sequences and simultaneously predict gene structures within both genomes.

Early large-scale genome projects relied heavily on the manual annotation of gene structures in order to ensure genome annotation of the highest quality [28-30]. Manual annotation involves scientists examining all of the evidence for gene structures as described above using a graphical genome viewer and annotation editor such as Apollo [31] or Artemis [32]. These manual efforts were, and continue to be, essential to providing the best community resources in the form of high quality and accurate genome annotations. Manual annotation is limited, though, because it is time consuming, expensive, and it cannot keep pace with the advances in high-throughput DNA sequencing technology that are producing increasing quantities of genome sequences.

FL-cDNA projects have lessened the need for manual curation of every gene by providing accurate and complete gene structure annotations derived from high-quality spliced alignments. Software such as Program to Assemble Spliced Alignments (PASA) [33] has enabled high-throughput automated annotation of gene structures by exploiting ESTs and FL-cDNAs alone or within the context of pre-existing annotated gene structures. Other, more comprehensive computational strategies have been developed to play the role of the human annotator by combining precomputed diverse evidence into accurate gene structure annotations. These tools include Combiner [34], JIGSAW [35], GLEAN [36], and Exogean [37], among others. These algorithms employ statistical or rule-based methods to combine evidence into a most probable correct gene structure.

We present a utility called EVidenceModeler (EVM), an extension of methods that led to the original Combiner development [34,38], using a nonstochastic weighted evidence combining technique that accounts for both the type and abundance of evidence to compute weighted consensus gene structures. EVM was heavily utilized for the genome analysis of the mosquito *Aedes aegypti *[39], and used partially or exclusively to generate the preliminary annotation for recently sequenced genomes of the blood fluke *Schistosoma mansoni *[40], the protozoan oyster parasite *Perkinsus marinus*, the human body louse *Pediculus humanus*, and another mosquito, *Culex pipiens*. The evidence utilized by EVM corresponds primarily to *ab initio *gene predictions and protein and transcript alignments, generated via any of the various methods described above. The intuitive framework provided by EVM is shown to be highly effective, exploiting high quality evidence where available and providing consensus gene structure prediction accuracy that approaches that of manual annotation. EVM source code and documentation are freely available from the EVM website [41].

## Results and discussion

In the subsequent sections, we demonstrate EVM as an automated gene structure annotation tool using rice and human genome sequences and related evidence. First, using the rice genome, we develop the concepts that underlie the algorithm of EVM as a tool that incorporates weighted evidence into consensus gene structure predictions. We then turn our attention to the human genome, in which we examine the role of EVM in concert with PASA to annotate protein-coding genes and alternatively spliced isoforms automatically. In each scenario, we include comparisons with alternative annotation methods.

### Evaluation of *ab initio *gene prediction in rice

**Figure 1:**
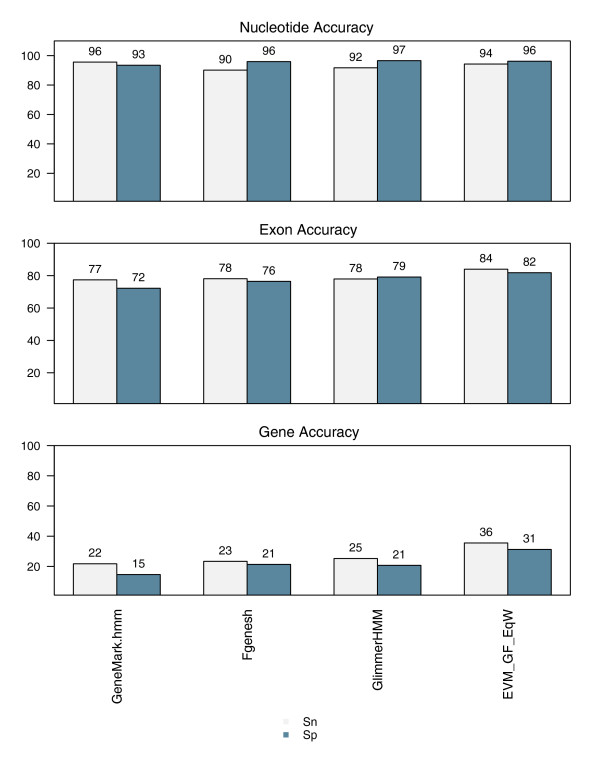
Rice *Ab initio *gene prediction accuracies. Gene prediction accuracies are shown for GeneMark.hmm, Fgenesh, and GlimmerHMM *ab initio *gene predictions based on an evaluation of 1058 cDNA-verified reference rice gene structures. The accuracy of EVidenceModeler (EVM) consensus predictions from combining all three *ab initio *predictions using equal weightings (weight = 1 for each) is also provided.

**Figure 2:**
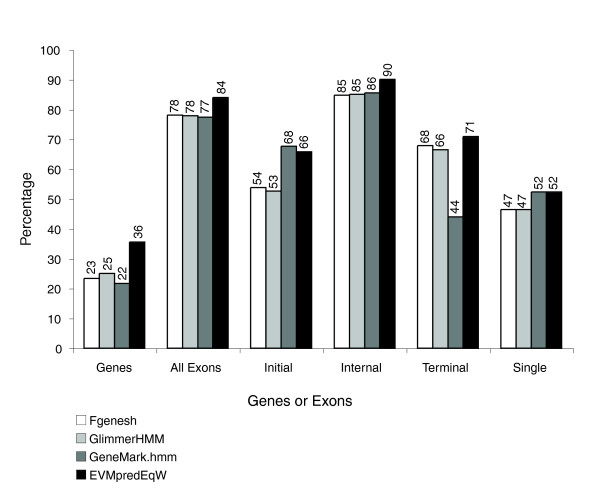
*Ab initio *prediction sensitivity by exon type. Individual *ab initio *exon prediction sensitivities based on comparisons with 1,058 reference rice gene structures are shown for each of the four exon types: initial, internal, terminal, and single. Results are additionally shown for EVidenceModeler (EVM) consensus predictions where the *ab initio *predictions were combined using equal weights.

The prediction accuracy for each of the three programs Fgenesh [5], GlimmerHMM [4], and GeneMark.hmm [6] was evaluated using a set of 1,058 cDNA-verified reference gene structures. All three were nearly equivalent in both their exon prediction accuracy (about 78% exon sensitivity [eSn] and 72% to 79% exon specificity [eSp]) and complete gene prediction accuracy (22% to 25% gene sensitivity [gSn] and 15% to 21% gene specificity [gSp]; Figure 1). The breakdown of prediction accuracy by each of the four exon types indicates that all gene predictors excel at predicting internal exons correctly (about 85% eSn) while predicting initial, terminal, and single exons less accurately (44% to 68% eSn; Figure 2).

**Figure 3:**
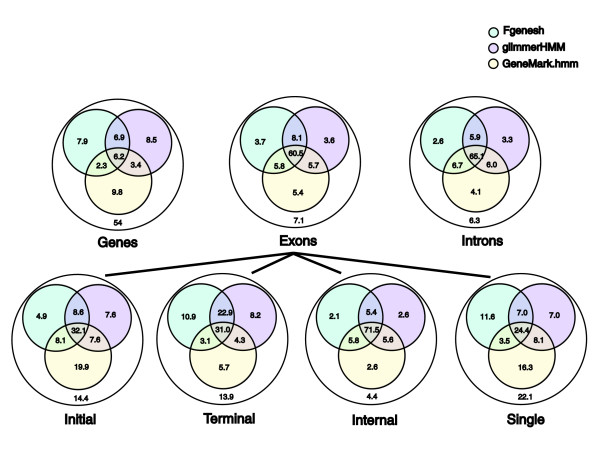
Venn diagrams contrasting correctly predicted rice gene structure components by *ab initio *gene finders. Percentages are shown for the fraction of 1,058 cDNA verified rice genes and gene structure components that were predicted correctly by each *ab initio *gene predictor. The cDNA-verified gene structure components consist of 7,438 total exons: 86 single, 5408 internal, 972 initial, and 972 terminal.

Although each gene predictor exhibits a similar level of accuracy, they differ greatly in the individual gene structures they each predict correctly. The Venn diagrams provided in Figure 3 reveal the variability among genes and exons predicted correctly by the three programs. Although each program predicts up to 25% of the reference genes perfectly, only about a quarter of these (6.2%) were identified by all three programs simultaneously. It is also notable that more than half (54%) of the cDNA-verified genes are not predicted correctly by any of the gene predictors evaluated. At the individual exon level, there is much more agreement among predictions, with 60.5% of the exons correctly predicted by all three programs. Only 7.1% of exons are not predicted correctly by any of the three programs. The Venn diagrams indicate much greater overall consistency among internal exon predictions, correlated with the inherently high internal exon prediction accuracy, as compared with the greater variability and decreased prediction accuracy among other exon types. A relatively higher proportion of the single (22.1%), initial (14.4%), and terminal (13.9%) exon types found in our reference genes are completely absent from the set of predicted exons.

### Consensus *ab initio *exon prediction accuracy

**Figure 4:**
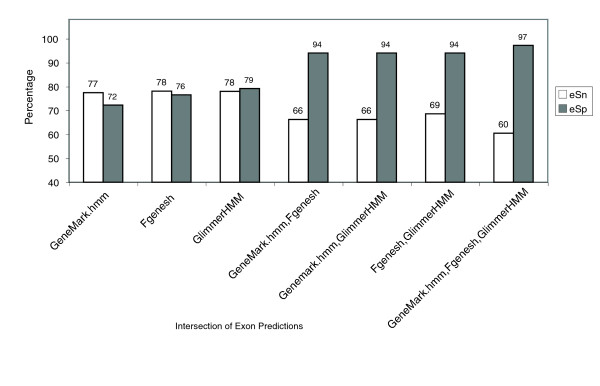
Exon prediction accuracy limited to consensus complete exon calls. Exon sensitivity (eSn) and exon specificity (eSp) were determined by comparing *ab initio *predicted exons. Exons were restricted to those perfectly agreed upon by either two or three different gene predictors. Only those predicted exons found within 500 base pairs flanking the 1,058 reference gene structures were considered for the specificity calculations.

Although there is considerable disagreement among exon calls between the various gene predictors, when multiple programs call exons identically they tend more frequently to be correct. Figure 4 shows that by restricting the analysis to only those exons that are predicted identically by two programs, exon prediction specificity jumps to 94% correct, regardless of the two programs chosen. Exon prediction specificity improves to 97% if we consider only those exons that are predicted identically by all three programs. Note that although the specificity improves to near-perfect accuracy, the prediction sensitivity drops from 78% to 60%. Although we cannot rely on shared exons to predict all genes correctly, we can in this circumstance trust those that are shared with greater confidence. EVM uses this increased specificity provided by consensus agreement among evidence for gene structure components and reports these specific components as part of larger complete gene structures; at the same time, EVM uses other lines of evidence to retain a high level of sensitivity.

### Consensus gene prediction by EVM

**Figure 5:**
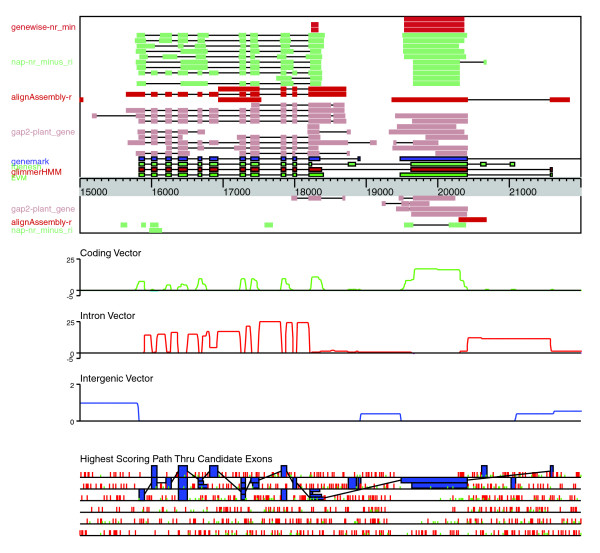
Consensus Gene Structure Prediction by EVM. The main aspects of the EVidenceModeler (EVM) weighted consensus prediction generating algorithm are depicted here, exemplified with a 7 kilobase region of the rice genome. The top view illustrates a genome browser-style view, showing the *ab initio *gene predictions GlimmerHMM, Fgenesh, and GeneMark.hmm, AAT-gap2 spliced alignments of other plant expressed sequence tags (ESTs), Program to Assemble Spliced Alignments (PASA) assemblies of rice EST and full-length cDNA (FL-cDNA) alignments, AAT-nap spliced alignments of nonrice proteins, and GeneWise protein homology-based predictions. Top strand and bottom strand evidence are separated by the sequence ticker. Evidence is dismantled into candidate introns and exons; candidate exons are shown in the context of the six possible reading frames at the figure bottom. A coding, intron, and intergenic score vector are shown; feature-specific scores (see Materials and methods) were added to corresponding vectors here for illustration purposes only, and note that all introns have feature-specific scores. The selection of exons, introns, and intergenic regions that define the highest scoring path is shown by the connections between exon features within the six-frame feature partition. This highest scoring path yields two complete gene structures, shown as an EVM tier at top, corresponding to the known rice genes (left) LOC_Os03g15860 (peroxisomal membrane carrier protein) and (right) LOC_Os03g15870 (50S ribosomal protein L4, chloroplast precursor).

Unlike conventional *ab initio *gene predictors that use only the composition of the genome sequence, EVM constructs gene structures by combining evidence derived from secondary sources, including multiple *ab initio *gene predictors and various forms of sequence homologies. In brief, EVM decomposes multiple gene predictions, and spliced protein and transcript alignments into a set of nonredundant gene structure components: exons and introns. Each exon and intron is scored based on the weight (associated numerical value) and abundance of the supporting evidence; genomic regions corresponding to predicted intergenic locations are also scored accordingly. The exon and introns are used to form a graph, and highest scoring path through the graph is used to create a set of gene structures and corresponding intergenic regions (Figure 5; see Materials and methods, below, for complete details). Because of the scoring system employed by EVM, gene structures with minor differences, such as small variations at intron boundaries, can yield vastly different scores. For example, a cDNA-supported intron that is only three nucleotides offset from an *ab initio *predicted intron could be scored extraordinarly high as compared with the predicted intron, although they differ only slightly in content. Likewise, an intron that is fully supported by multiple spliced protein alignments will be scored higher than an alternate intron of similar length yielded by only a single similarly weighted protein alignment. In this way, EVM uses the abundance and weight of the various evidence to score gene structure components appropriately to promote their selection within the resulting weighted consensus genome annotation.

To demonstrate the simplest application of EVM, we combine only the three *ab initio *gene predictions and weight each prediction type equally. Figures 1 and 2 display the results in comparison with the *ab initio *prediction accuracies; we demonstrate that, by incorporating shared exons and introns into consensus gene structures, complete gene prediction accuracy is improved by at least 10%. Exon prediction accuracy is increased by about 6%, and exon prediction accuracies for each exon type are mostly improved, with the exception of the initial exon type, for which GeneMark.hmm alone is slightly superior.

### Consensus gene prediction accuracy using varied evidence types and associated weights

**Figure 6:**
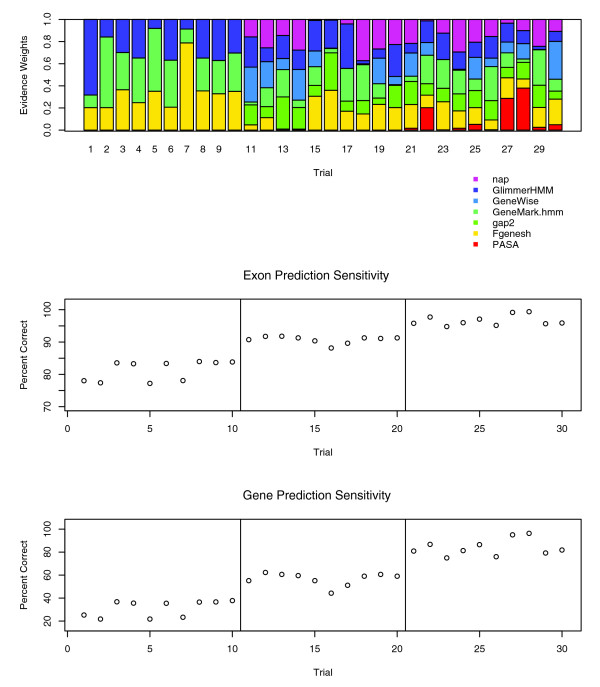
Response of EVM prediction accuracy to varied evidence types and weights. Iterations (30) of randomly weighted evidence types were evaluated by EVidenceModeler (EVM). Iterations 1 to 10 included only the *ab initio *predictors GlimmerHMM, Fgenesh, and GeneMark.hmm. Iterations 11 to 20 additionally included AAT-nap alignments of nonrice proteins, GeneWise predictions based on nonrice protein homologies, and AAT-gap2 alignments of other plant expressed sequence tags. Iterations 21 to 30 included Program to Assemble Spliced Alignments (PASA) alignment assemblies and corresponding supplement of PASA long-open reading frame (ORF)-based terminal exons. Exon and complete gene prediction sensitivity values resulting from EVM using the corresponding weight combinations are plotted below.

A gene structure consensus as computed by EVM is based on the types of evidence available and their corresponding weight values. In the example above, each evidence type provided in the form of *ab initio *gene predictions was weighted identically. In the case where each prediction type is equivalent in accuracy, this may be sufficient, but when an evidence type(s) is more accurate, a higher weight(s) applied to that evidence is expected to drive the consensus toward higher prediction accuracy. Figure 6 illustrates the impact of varied weight combinations and sources of evidence on exon and complete gene structure prediction sensitivity. In the first set (iterations 1 to 10), only the three *ab initio *gene predictions are combined using random weightings. Prediction accuracy ranges from 22% to 38% gSn and 77% to 84% eSn. In the second set (iterations 11 to 20), sequence homologies are additionally included in the form of spliced protein alignments (using nap of AAT), spliced alignments of ESTs derived from other plants (using gap2 of AAT), and GeneWise protein-homology-based gene predictions. There, complete prediction accuracy ranges from 44% to 62% gSn and 88% to 92% eSn. In the third and final set (iterations 21 to 30), PASA alignment assemblies derived from rice transcript alignments were included, from which a subset define the correct gene structure. In the presence of our best evidence and randomly set weights, prediction accuracy ranges from 75% to 96% gSn and 95% to 99% eSn.

Although this represents just a minute number of possible random weight combinations, it demonstrates the effect of the weight settings and the inclusion of different evidence types on our consensus prediction accuracy. By including evidence based on sequence homology, our prediction accuracy improves greatly, doubling to tripling complete gene prediction accuracy of *ab initio *programs alone or in combination. Also, very different weight settings can still lead to similar levels of performance, particularly in the presence of sequence homology data.

### EVM consensus prediction accuracy using trained evidence weights

Given the variability in consensus gene prediction accuracy observed using different combinations of weight values, finding the single combination of weights that provides the best consensus prediction accuracy is an important goal. Searching all possible weight combinations to find the single best scoring combination is not tractable, given the computational effort needed to explore such a vast search space. To estimate a set of high scoring weights, we employed a set of heuristics that use random weight combinations followed by gradient ascent (see Materials and methods, below). For the purpose of choosing high performing weights and evaluating their accuracy, we selected 1,000 of our cDNA-verified gene structures and used half for estimating weights and the other half for evaluating accuracy using these weights (henceforth termed 'trained weights'). In both the training and evaluation process, accuracy statistics were limited to each reference gene and flanking 500 base pairs (bp). However, EVM was applied to regions of the rice genome including the 30 kilobase (kb) region flanking each reference gene, to emulate gene prediction by EVM in a larger genomic context.

**Figure 7:**
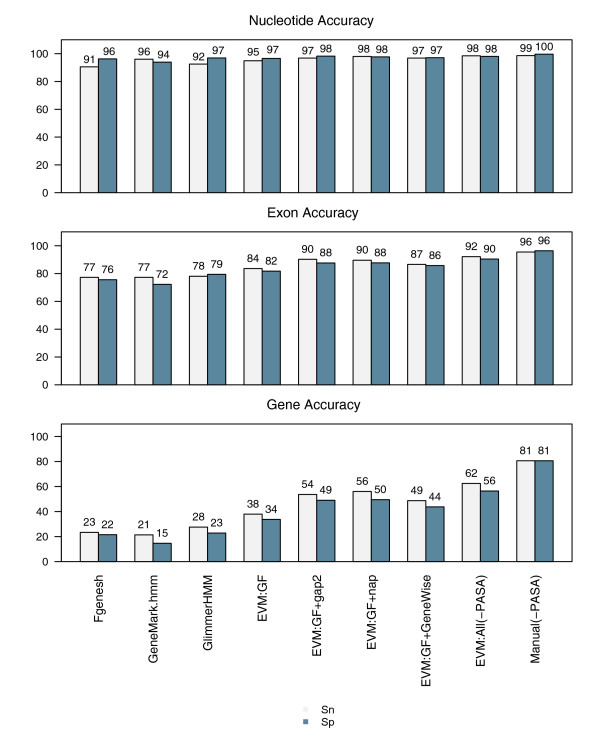
Rice consensus gene prediction accuracy using optimized evidence weights. Gene prediction accuracy for EVidenceModeler (EVM) was calculated at the nucleotide, exon, and complete gene level using trained weights and specific sets of evidence, applied to 500 of the reference rice gene structures. The evidence evaluated is described as follows: EVM:GF includes *ab initio *gene predictions (GF) alone; EVM:GF+gap2 includes GF plus the AAT-gap2 alignments of other plant expressed sequence tags (gap2); EVM:GF+nap includes GF plus AAT-nap alignments of nonrice proteins (nap); EVM:GF+GeneWise includes GF plus the GeneWise predictions based on nonrice protein homologies (GeneWise); EVM:ALL(-PASA) includes GF, nap, gap2, and GeneWise; EVM:ALL(+PASA) additionally includes the Program to Assemble Spliced Alignments (PASA) alignment assemblies and PASA long-open reading frame (ORF)-based terminal exon supplement. Sn, sensitivity; Sp, specificity.

Because the training of EVM is not deterministic, and each attempt at training can result in a different set of high-scoring weights, we performed the process of training and evaluating EVM on the rice datasets three times separately. The trained weight values computed by each training process are provided in Additional data file 2 (Table S1), and the consensus gene prediction accuracy yielded during each evaluation is provided in Additional data file 2 (Table S2). The average gene prediction accuracy is provided in Figure 7. On this set of 500 reference genes, the average exon and complete gene prediction accuracies for the *ab initio *predictors are similar to those computed earlier for the larger complete set of 1,058 cDNA-verified genes. EVM applied to the *ab initio *predictions alone using optimized weights yielded 38% gSn and 34% gSp, approximately 10% better than the best corresponding *ab initio *accuracy. By including the additional evidence types in the form of protein or EST homologies independently, complete gene prediction sensitivity increases to 49% to 56% gSn and 44% to 50% gSp. Using all evidence minus the PASA data, complete gene sensitivity reaches 62% gSn and 56% gSp. Note that each gain in sensitivity is accompanied by a gain in specificity, indicating overall improvements in gene prediction accuracy.

### Intuitive versus trained weights

Although we can computationally address the problem of finding a set of weights that yield optimal performance, it is clear from our analysis of randomly selected weights that there could be numerous weight combinations that provide reasonable accuracy. In general, we find that combinations of assigned weightings in the following form provides adequate consensus prediction accuracy:

(*ab initio *predictions) ≤ (protein alignments, EST alignments) < (GeneWise) < (PASA)

Using such a weight combination (gene predictions = 0.3, proteins and other plant ESTs = 1, GeneWise = 5, PASA = 10), we find that our consensus exon and complete gene prediction accuracy is quite comparable, with our intuitive weights providing performance levels that in most cases are just slightly lower than those of our trained weights (Additional data file 1 [Figure S1]). In each case, accuracy measurements with intuitive weight settings were within 3% of the results from trained weights. The ability to tune EVM's evidence weights intuitively provides a flexibility that is not as easily afforded by current software systems based on a strict probabilistic framework.

### EVM versus alternative annotation tools: Glean and JIGSAW

**Figure 8:**
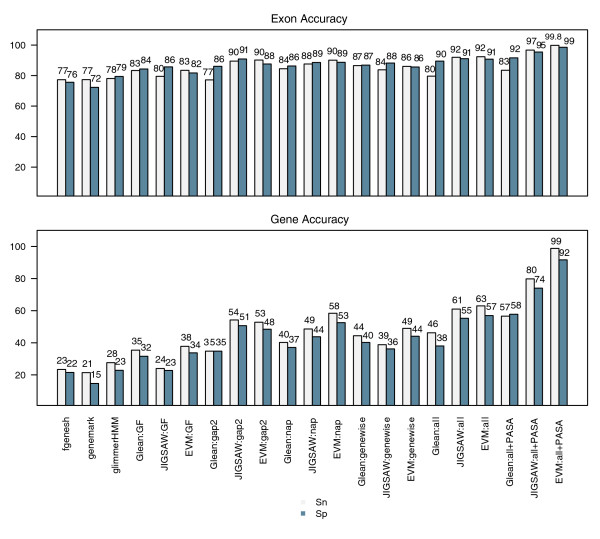
EVM's accuracy compared with Glean and JIGSAW. Both JIGSAW and Glean were trained and evaluated on the rice genome data, and accuracies were compared with those of EVidenceModeler (EVM). The trained weights utilized by EVM are provided in Additional File 2 (Table S3). PASA, Program to Assemble Spliced Alignments; Sn, sensitivity; Sp, specificity.

The accuracy of EVM was compared with that of competing combiner-type automated annotation tools using both Glean and JIGSAW. The publicly available Glean and JIGSAW software distributions were downloaded and run using default parameter settings. We trained JIGSAW using datasets identical to those provided to EVM, using the 500 reference genes and associated evidence for training and the separate 500 genes and evidence for evaluation. Glean's unsupervised training is tightly coupled to the prediction algorithm, and so Glean was executed on the entire set of 1,000 genes and associated evidence, with the proper half used for evaluation purposes. Exon and complete gene prediction accuracies are shown in Figure 8. Each evidence combiner demonstrates substantial improvements in accuracy in the presence of sequence homology evidence. EVM fares well in this combiner showdown, and in most cases it provides the greatest prediction accuracy of the three tools analyzed.

The prediction accuracy between JIGSAW and EVM is strikingly similar for two of the evidence combing scenarios examined: combining gene predictions with other plant EST alignments (gap2), and when all alignment data are included minus the rice PASA evidence (all). We further examined the latter case, in which both JIGSAW and EVM predicted more than 60% of the complete genes accurately, to determine the similarity of their gene predictions. Of the 500 reference genes tested, there are 310 predictions generated identically between EVM and JIGSAW, of which 260 were correct. Therefore, although their prediction accuracies can be strikingly similar, overall the gene structures predicted are quite different.

A strength of EVM is its ability to utilize heavily trusted forms of evidence, such as gene structures inferred from alignments of cognate FL-cDNAs and ESTs. Each of the three programs were trained in the presence of cDNA-supported gene structures as provided by PASA (long open reading frame [ORF] structures within PASA alignment assemblies), a subset of that defines a correct gene structure (see Materials and methods, below). All three tools demonstrated the greatest prediction accuracy in the presence of PASA evidence. Although each tool is effectively provided with evidence containing all complete introns and exons that define the correct gene structure, only EVM is found to be capable of nearly perfect prediction accuracy. Of the 500 evaluated reference genes, EVM predicted only six incorrectly when supplied with PASA evidence along with the competing evidence types (*ab initio *predictions, and protein and other plant EST alignments). These six incorrect predictions involved three cases in which neighboring genes were merged into single predictions, two cases in which improper gene termini were chosen, and a single case that was confounded by a large degenerate retrotransposon insertion within an intron of a gene, an element that was not masked and excluded from the gene prediction effort.

### Comparison with manual annotation

It is expected and reassuring that EVM provides nearly perfect complete gene accuracy in the presence of high quality and reliable complete gene structure data, as provided in the form of the PASA alignment assemblies. The importance of such ESTs and FL-cDNAs for gene structure annotation is well known [42-45], and software such as PASA can annotate gene structures based solely on these data in absence of pre-existing gene annotations or *ab initio *gene predictions [33]. A greater challenge is to achieve maximal consensus gene prediction accuracy in the absence of these data, which is the typical scenario with newly sequenced genomes that lack extensive EST or FL-cDNA sequences as companion resources. In such cases we must rely on the accuracy of *ab initio *gene predictors and homologies to sequences from other organisms, and it is here that, in lieu of an equivalent automated annotation method, we expect to have the greatest gains from expert scientists directly evaluating and modeling complete gene structures based on these sources of evidence.

In our application of EVM thus far, the relevant set of input evidence is that which contains the *ab initio *gene predictions, protein alignments, GeneWise predictions based on protein homology, and the alignments to ESTs derived from other plants (Figure 7; entry 'EVM:All(-PASA)', read as EVM with all evidence minus PASA evidence). Using trained weights, EVM correctly predicted 92% of the known exons and 62% of the 500 cDNA-verified genes correctly, on average. If the subset of the native cDNA data that defines the correct gene structure is not supplied as evidence, and if components of such known gene structures are not available as candidate introns and exons, then EVM will be unable to predict the gene correctly. In an effort to establish the upper limit of gene prediction accuracy in the absence of cDNA evidence, we propose use of the accuracy of manual annotation on the same dataset. The accuracy of human annotation has never been adequately measured, although it is widely assumed that human annotation is the 'gold standard' for genome projects. For our study, a set of human annotators was asked to evaluate these data in absence of cognate rice cDNA alignments, and were instructed to model a gene structure manually that best reflected the available evidence. In absence of the rice cDNAs, manual annotation accuracy resulted in 96% eSn and 96% eSp, and 81% gSN and 81% gSP (Figure 7). In light of these statistics, we consider the accuracy provided by EVM on the identical dataset to be demonstrably effective as an automated annotation system, and approaching the better accuracy obtained through manual curation efforts, particularly when compared with the accuracy of individual *ab initio *gene predictors on the same dataset.

### Application of EVM and PASA to the ENCODE regions of the human genome

The ENCyclopedia of DNA Elements (ENCODE) project was initiated shortly after the sequencing of the human genome with the aim being to identify all functional elements, including all protein-coding genes, in the human genome sequence [46]. The pilot phase of the project focused on only 1% (about 30 megabases spread across 44 regions) of the genome, termed the ENCODE regions. The GENCODE (encyclopedia of genes and genes variants) consortium was formed to provide high quality manual annotation and experimental verification of protein coding genes in these regions [47]. The human ENCODE Genome Annotation Assessment Project (EGASP) was established to evaluate the accuracy of automated genome annotation methods by comparing automated annotations of the ENCODE regions with the GENCODE annotations [10]. Participants in the EGASP competition were allowed access to 13 ENCODE regions along with their corresponding GENCODE annotations, which could be used for training purposes. Groups submitted their automated annotations for the remaining 31 regions, after which time the corresponding GENCODE annotations were released and the automated annotation methods were evaluated based on a rigorous comparison with the GENCODE annotations [48].

The sequences, gene predictions, and annotations involved in EGASP additionally serve as a resource for evaluating current and future annotation methods. Similarly to our application of EVM to the rice genome using cDNA-verified gene structures for training and evaluation purposes, we applied EVM to the ENCODE regions using the GENCODE annotations for training and evaluation purposes, analogous to the original EGASP competition. Evidence used by EVM included the evidence tracks provided by University of California at Santa Cruz: TWINSCAN, SGP2, GENEID, GENSCAN, CCDSGene, KNOWNGene, ENSEMBL (ENSGene), and MGCGene. Additional evidence generated in our study included AAT alignments of nonhuman proteins, GeneWise predictions based on the nonhuman protein homologies, AAT nucleotide alignments of select animal gene indices, and PASA alignment assemblies generated from GMAP alignments of human ESTs and FL-cDNAs. The GlimmerHMM predictions used by EVM were those generated as part of the EGASP competition, and were obtained separately.

There are several notable differences between the training and evaluation of EVM on the ENCODE regions as compared with the earlier application to rice. The cDNA-verified rice genes used for training and evaluation were restricted to a single splicing isoform. In addition, each gene was complete, containing the protein-coding region from start to stop codon. The GENCODE protein-coding annotations, in contrast, include alternative splicing isoforms and several partial gene structures. Accuracy measurements computed for rice genes included each cDNA-verified gene and the flanking 500 bases, whereas accuracy measurements on the ENCODE regions included these sequence regions in their entirety and all corresponding protein-coding gene annotations.

**Figure 9:**
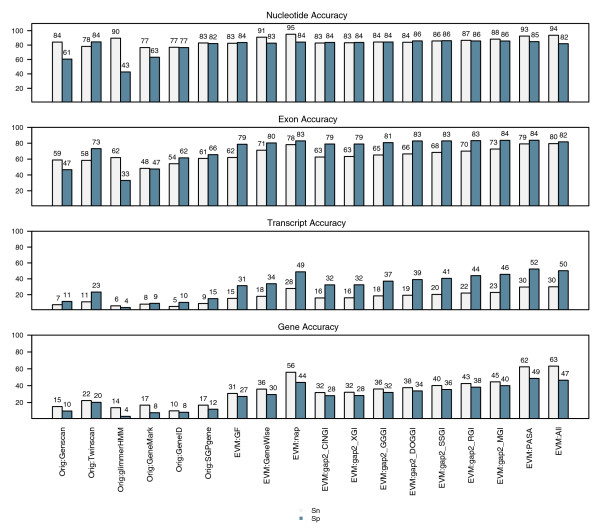
Human consensus gene prediction accuracy by EVM. The consensus gene prediction accuracy by EVidenceModeler (EVM) is shown based on trained evidence weights and the corresponding combination of evidence, as applied to the GENCODE test regions of the human genome. The accuracies for the inputted gene predictions obtained from the ENCODE Genome Annotation Assessment Project (EGASP) dataset are provided for reference sake, including GENSCAN, TWINSCAN, GlimmerHMM, GeneMark.hmm on the repeat-masked genome, GeneID, and SGPgene. EVM-GF corresponds to EVM applied to these gene prediction tiers alone (GF), and serves as the baseline evidence for the subsequent entries. EVM-GeneWise includes GeneWise predictions based on nonhuman protein homologies; EVM-nap includes AAT-nap spliced alignments of nonhuman proteins; the EVM:gap2_* series includes AAT-gap2 alignments of corresponding transcripts from the Dana Farber Gene Indices (CINGI, *Ciona intestinalis *[sea squirt]; XGI, *Xenopus tropicalis *[frog]; GGGI, *Gallus gallus *[chicken]; DOGGI, *Canis familiaris *[dog]; SSGI, *Sus scrofa *[pig]; RGI, rat; MGI, mouse); EVM-alignAsm includes Program to Assemble Spliced Alignments (PASA) alignment assemblies and corresponding terminal exon supplement; and EVM:All includes all evidence described (GF, gap2, nap, GeneWise, and PASA). Sn, sensitivity; Sp, specificity.

EVM was trained on the 11 ENCODE test regions and then evaluated on the remaining 33 regions. Training and evaluation were performed under two independent trials. The trained weights and corresponding accuracy values are provided in Additional data file 2 (Tables S4 and S5). Our initial analysis of EVM on this dataset utilized the *ab initio *gene predictions, and the EST and protein homologies, similar to our earlier analysis with rice. The average gene prediction accuracy for the source predictions and EVM with varied additional evidences is illustrated in Figure 9. The *ab initio *gene predictions used as evidence by EVM individually predict genes with accuracies mostly less than 20% gSn; the best individual performer was TWINSCAN, with 22% gSn and 20% gSp. By combining these predictions alone, EVM improves complete gene prediction accuracy to 31% gSn and 27% gSp, which is significantly better performance than any of the individual *ab initio *predictors. By including spliced alignments to dog, pig, mouse, or rat assembled EST databases, gene prediction sensitivity further improves to 38% to 45% gSn and 34% to 40% gSp. EST alignments from the more distantly related chicken yield slight improvement from using the predictions alone, but not to the extent of mammals. Alignments to the more distantly related sea squirt and frog gene indexes offer little to no improvement in prediction accuracy. Overall, the improvements in EVM prediction accuracy afforded by alignments to the nonhuman gene indexes correlate well with their phylogenetic distance from human, with mouse and rat being found most useful. By including human EST and FL-cDNA alignments in the form of PASA alignment assemblies along with the *ab initio *predictions, gene prediction sensitivity improves to 63%. Protein homologies included with *ab initio *predictions, in the form of AAT (nap) alignments or GeneWise predictions, also demonstrated an improvement in gene prediction accuracy, with 36% to 56% gSn and 30% to 44% gSp as compared with the 31% gSn and 27% gSp from combining the predictions alone.

### Post-EVM application of PASA to annotate alternatively spliced isoforms

EVM is not designed to model alternative splicing isoforms directly. This is, however, a primary function of our companion annotation tool PASA, which contributes to the automated annotation of gene structures in several ways. PASA, like EVM, is made freely available as open source from the PASA website [49]. Above, PASA alignment assemblies were used as one source of gene structure components by EVM. Alternatively, PASA can generate complete gene structures based on full-length alignment assemblies (alignment assemblies containing at least one FL-cDNA) by locating the longest ORF within each alignment assembly, and annotate gene structures and alternatively spliced isoforms restricted to the transcriptome. A third application of PASA is to perform a retroactive processing of a set of pre-existing gene structure annotations, whereby alignment assemblies are incorporated into untranslated region annotations, exon modifications, correctly splitting or merging predicted gene structures, and used to model alternative splicing isoforms [33].

To demonstrate the effect of applying PASA as a postprocess to integrate transcript data into an existing set of gene structure annotations (which we refer to as 'PASAu', for PASA updates), we applied PASA separately to the *ab initio *predictions, the various University of California at Santa Cruz gene prediction tracks (which we refer to as 'other predictions'), and to the EVM-generated datasets that either utilized or excluded the other predictions. The change in prediction accuracy as a result of applying PASA's annotation updates is illustrated in Additional data file 1 (Figure S2). PASAu can yield relatively large improvements (increases from 23% to 33% in gSn and from 7% to 32% in gSp) to the accuracy of the various *ab initio *predictions by incorporating transcript alignment assembly-based updates. PASAu-resulting changes to the accuracies of the other original predictions were more variable, mostly involving small increases in transcript sensitivity and larger decreases in transcript specificity; more GENCODE transcripts predicted correctly, but additional PASA-based transcripts not represented in the GENCODE dataset were also identified. The EVM gene sets were affected similarly.

**Figure 10:**
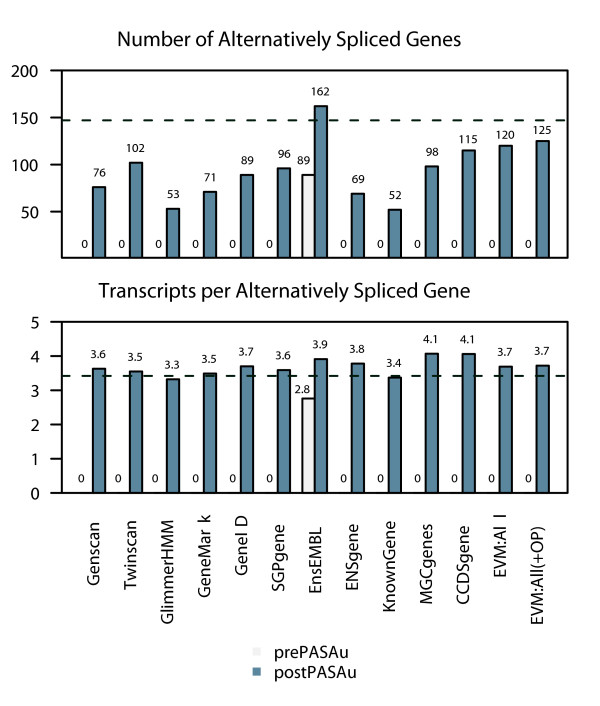
Addition of alternatively spliced isoforms using PASAu. By applying Program to Assemble Spliced Alignments (PASA) to the various annotation datasets, PASA can automatically annotate alternative splicing isoforms. The number of alternatively spliced genes and the number of transcripts per alternatively spliced gene are shown, including the pre-PASAu and post-PASAu values. Only the EnsEMBL dataset includes models for alternatively spliced isoforms before the application of PASA. Dotted lines indicate the corresponding values based on the GENCODE reference annotation dataset: 147 alternatively spliced genes and 3.42 transcripts per alternatively spliced gene. Transcript isoforms alternatively spliced only in untranslated regions were ignored. Here, EVM:All(+OP) refers to the inclusion of the EVM:All evidence plus the 'other predictions' from ENCODE Genome Annotation Assessment Project (EGASP), including EnsEMBL, ENSgene, KnownGene, and CCDSgene, used by EVidenceModeler (EVM) as the OTHER_PREDICTION evidence class (Table 1).

The small change in gSn and gSp resulting from the annotation update functions of PASA to the EVM predictions is not surprising, given that the PASA alignment assemblies were included here as inputs during the generation of the consensus gene structures by EVM. The most notable consequence of the PASA updates was the modeling of alternative splicing isoforms. Although the number of genes annotated as alternatively spliced was variable across the different annotation gene sets, the ratio of transcripts per alternatively spliced gene was fairly uniform, and largely consistent with the prevalence of alternatively spliced genes described in the GENCODE annotations (Figure 10). The reason for the variability in the number of alternatively spliced genes is because of PASAu's stringent validation tests, forsaking automated gene structure updates in favor of targeted manual evaluation in those cases in which the tentative gene structure updates or candidate splicing isoforms vary greatly from the originally annotated gene structures [49].

**Figure 11:**
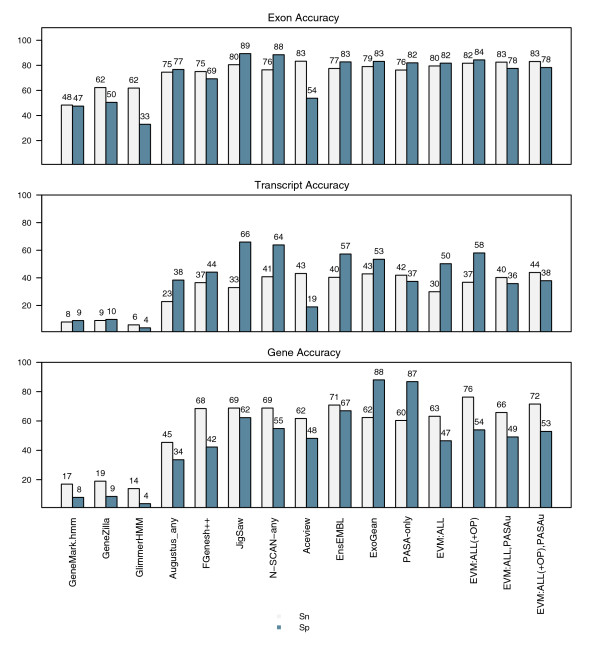
EVM and PASA automated annotation accuracies compared to alternatives. The gene prediction accuracy of both EVidenceModeler (EVM) and Program to Assemble Spliced Alignments (PASA) are shown in the context of the other methods evaluated as part of the ENCODE Genome Annotation Assessment Project (EGASP) competition. Although PASA alone performs quite well, the benefits from applying PASA as a postprocess to the EVM consensus predictions are not immediately apparent, except in the enumeration of alternatively spliced isoforms as shown in Figure 10. PASA and EVM are shown to perform similarly to the best performing methods in the EGASP competition.

The gene prediction accuracy of EVM, PASA alone, and PASA applied as a postprocess to update EVM predictions is provided along with the accuracies of methods evaluated as part of the EGASP competition in Figure 11. PASA, when used in isolation to annotate gene structures automatically based on transcript alignments alone, yields an impressive 60% gSN and 87% gSP; these values reflect the abundance and utility of the human ESTs and FL-cDNAs available. EVM, with its greatest accuracy throughout the various surveys of the EGASP dataset presented, yielded prediction accuracies of between 63% and 76% gSn and of between 47% to 54% gSp.

Although it is useful to compare accuracies of these various tools based on their ability to recreate the GENCODE annotation for the ENCODE regions, direct comparisons between each method based on these data may be generally useful but not exactly valid. In the case of *ab initio *gene prediction tools that require only the genome sequence as input, direct comparisons between the results of the gene predictors are fully justified, because the inputs are exactly identical. The focus of EGASP was to examine the accuracy of diverse automated annotation methods and not necessarily to perform head-to-head comparisons between each method. Therefore, groups were allowed to use any evidence available to them to assist in their annotation efforts, and so, for example, the additional evidence used by JIGSAW was not exactly the same inputs utilized by Exogean, or EVM as described here. The analogous experiments we directed in rice were more tightly controlled, given that each software tool was trained and executed using identical inputs. Even so, although alternative methods examined as part of the EGASP competition are shown to exceed EVM's accuracy, even if only slightly, EVM does fare well as an automated annotation system, especially when it is compared with the individual *ab initio *predictions.

## Conclusion

We have demonstrated that EVM is an effective automated gene structure annotation tool that leverages *ab initio *gene predictions and sequence homologies to generate weighted consensus gene predictions. The gene prediction accuracy of EVM is influenced by the types of evidence provided and associated weight values. Although a training system is provided to assist the search for optimal evidence weights, a manually set weighting scheme can perform similarly. We demonstrated the general utility of EVM as an automated annotation utility using both rice and human genome sequences. We also showed how to use PASA to provide an effective postprocessing step to discover and annotate alternatively spliced isoforms. EVM, especially when combined with PASA, provides an intuitive and flexible automated eukaryotic gene structure annotation framework, reducing the manual effort required to produce a high quality and reliable gene set to support the earliest efforts of furthering our scientific understanding of the genome biology of eukaryotes. Both EVM [41] and PASA [49] are fully documented and freely available as open source from their respective websites.

## Materials and methods

### Generating evidence for gene structures

The *ab initio *gene prediction programs Fgenesh [5], GeneMark.hmm [6], and GlimmerHMM [4] were applied to the rice genome sequences. Fgenesh and GlimmerHMM were applied to repeat-masked genome sequences. Repeats were masked using RepeatMasker [50] and the rice repeat library [51]. GeneMark.hmm was applied to the unmasked genome sequence; software problems prevented us from running GeneMark.hmm on all repeat-masked genome sequences, and so we chose instead to use the unmasked genome in this case. The AAT software [12] was used to generate spliced protein and transcript alignments. For generating spliced protein alignments, AAT was used to search a comprehensive and nonredundant protein database that was first filtered from rice protein sequences. A database of other plant transcript sequences was compiled by downloading and joining all plant gene indices provided by The Gene Index at the Dana Farber Cancer Institute [52], excepting the rice gene indices. Rice ESTs and FL-cDNAs were aligned to the rice genome and assembled into gene structures as described previously [53], with the exception being that the high quality single-exon transcript alignments were included here along with spliced alignments.

### Compiling a reference rice gene set

We extracted PASA assemblies encoding a complete ORF exceeding 100 amino acids and considered these as candidates for high confidence complete gene structures, first requiring manual verification. For the purpose of training and evaluating EVM, we sought approximately 1,000 total high confidence gene structures, half to be used for training and the remainder for evaluation. In an effort to select this subset of genes, we manually examined the candidate PASA-based structures in the context of the available evidence using the TkGFF3 graphical genome viewing utility provided in the EVM software distribution. We then selected PASA-based structures that appeared to provide the best gene structure as the reference gene structures, yielding 1,058 such genes. We excluded PASA assemblies found to harbor rare AT-AC introns, to encode less than full-length ORFs, or to represent splicing variants that did not best represent the additional evidence. These excluded assemblies comprised approximately 10% of the total. To simplify training and evaluation of EVM, we extracted each high confidence gene and flanking 30 kb region from the complete rice genome and prepared these as independent and individual datasets.

All sequences, gene structures, and evidence are available for download [41]. A comparison of the distribution of coding exon counts among the gene structures in the training set as compared with all candidates and the release-4 gene structure annotations (non-TE set) is provided in Additional data file 1 (Figure S3). Although our verified set of known gene structures is notably deficient in single-exon genes, overall it is consistent with the other selections of rice genes and deemed suitable for our purposes herein.

### GENCODE annotations for ENCODE regions

We obtained the ENCODE region sequences, GENCODE annotations, and the various EGASP annotation datasets from the EGASP ftp site [54]. We encountered some difficulties working with the downloaded data files because of inconsistent file formats, inconsistent annotation of stop codons, and annotation features extending out of the sequence range. We therefore converted each data file over to a more strict GTF format, clipping annotations at the bounds of the ENCODE regions and adding stop codons where they were obviously lacking. Prediction accuracies of the EGASP datasets were recomputed (Additional data file 1 [Figure S4]) and were found to agree with the previously reported values; small differences between our recomputed values and previously published values are likely because of the slight differences in our stated implementation of our accuracy evaluation software and those differences resulting from our file conversions. Our refined versions of the EGASP datasets are available from the EVM software website [41].

Additional evidence compiled for the GENCODE annotations included homologies to nonhuman proteins using AAT-nap and GeneWise, alignments to assembled animal ESTs downloaded from the Gene Index using AAT-gap2, and PASA alignment assemblies. This additional evidence is also available from the EVM software site [41].

### EVM algorithm

EVM reports consensus gene structures as high scoring paths through a directed acyclic graph containing complete intron, exon, and intergenic region features as vertices. Each of the possible features is computed based on the evidence provided in the form of the genome sequence, *ab initio *gene predictions, and the transcript and protein alignments. Each type of evidence, such as the name of the gene prediction program or the combination of alignment method and sequence database searched, has an associated numeric weight value. This weight value is either set by hand or by the training process described below. The evidence and corresponding weights are used to score the exon, intron, and intergenic region features. Consensus gene structures reported by EVM are computed by connecting exons, introns, and intergenic regions across the complete genome sequence such that the series of connected components provides the highest cumulative score. An example of EVM applied to a section of the rice genome, including components of the scoring system and feature set, is illustrated in Figure 5. For large genome sequences (>1 megabase), the data are partitioned into overlapping segments, and the EVM predictions from the separate partitions are subsequently joined into a single nonredundant set of predictions.

### Dismantling predictions and alignments into exons and introns

Exons of eukaryotic gene structures are commonly treated as four distinct types: initial exon, including the start codon to a donor splice junction; internal exon, including an acceptor splice junction to a donor splice junction; terminal exon, including the acceptor splice junction to the stop codon; and the single exon, which corresponds to an intronless gene from start codon to stop codon. These are the four types of exons considered by EVM. The *ab initio *gene predictions provided as inputs to EVM are dismantled into their component exons and introns and added to a nonredundant corresponding exon or intron feature set. Each exon of a given type is stored by EVM with its coordinates, the codon position of its leading base, and a list of all evidence types that perfectly support it. Introns are likewise stored as discrete features based on unique coordinate pairs and their supporting evidence. Only the consensus GT or GC donor and AG acceptor dinucleotide splice sites are treated as valid by EVM; the more rare AT-AC consensus introns, although accepted by PASA, are currently disallowed by EVM. No maximum intron length is enforced by EVM, but a minimum intron length of 20 bp is set and can be tuned as required.

Protein and transcript spliced alignment inputs to EVM, by default, are only capable of contributing internal exons and introns to EVM's feature set. Spliced alignments contribute internal exons to the feature set for those internal alignment segments that have consensus splice sites and encode an ORF in at least one of the three reading frames. An internal exon is added to the feature set for each incident codon position that provides an ORF on that strand. A final way for alignment data to contribute initial, terminal, or single exons to the feature set is by explicitly providing such candidate exons to EVM *a priori*. This is one mechanism that allows EVM to better exploit gene structures provided by PASA. PASA includes functions to provide the longest ORF within each PASA assembly, and EVM includes a utility that extracts initial, terminal, and single exons from gene structures corresponding to the longest ORF within each PASA assembly. This list of PASA-based exon candidates can be provided directly to EVM. Internal exons provided by PASA alignment assemblies are included in the feature set exactly as other forms of spliced alignment data described above.

Experiments performed on the rice genome utilizing PASA evidence as input instead included the structure of the longest ORF (minimum length of 50 amino acids) within each PASA alignment assembly in place of the alignment assemblies themselves supplemented with the terminal exon candidates, as described above. These PASA longest ORF structures were provided to EVM as an OTHER_PREDICTION evidence class. Utilization of the PASA data in this way was necessary to allow provision of identical PASA-based evidence to the alternative annotation tools Glean and JIGSAW as part of the rice combiner accuracy comparison.

### Scoring genome features

The candidate unique exon, intron, and intergenic region feature types derive their score from either a feature-specific score and/or a corresponding feature type scoring vector, as described below. Each type of evidence provided to EVM is specified as having a numerical weight value and belonging to one of the four allowable classes: PROTEIN, TRANSCRIPT, ABINITIO_PREDICTION, or OTHER_PREDICTION. Table 1 indicates the scoring mechanism for each feature type and classification. Primary differences between these four classes of evidence are that the PROTEIN and TRANSCRIPT classes are not expected to encode complete gene structures from start to stop codon, but instead contribute components of gene structures such as internal exons and, in the case of the PROTEIN class, an indication of coding nucleotides. Complete gene predictions are partitioned into the classes ABINITIO_PREDICTION and OTHER_PREDICTION, where the ABINITIO_PREDICTION class predicts noncoding intergenic regions (GeneMark.hmm) and OTHER_PREDICTION allows for the inclusion of high-specificity forms of complete predictions that are not intended to delineate the noncoding intergenic regions (KnownGene).

**Table 1:** EVM scoring mechanism based on feature class and type

Class	Type	Scoring vector	Feature-specific score
ABINITIO_PREDICTION	Exon	X	
ABINITIO_PREDICTION	Intron		X
ABINITIO_PREDICTION	Intergenic	X	
TRANSCRIPT	Exon	X	
TRANSCRIPT	Intron		X
PROTEIN	Exon	X	
PROTEIN	Intron		X
OTHER_PREDICTION	Exon		X
OTHER_PREDICTION	Intron		X

EVM, EVidenceModeler.

A feature type scoring vector contains a numerical value for each nucleotide across the genome sequence. Evidence that contributes to a feature type scoring vector contributes its corresponding weight value to each nucleotide within the span of its feature coordinates. Evidence that contributes a feature-specific score instead contributes a value of its (weight × feature_length) to that unique feature that it supports, in this case either that complete intron or exon. Exons derive their scores from a combination of feature-specific scores and a corresponding scoring vector. In this case, the feature-specific scores are summed with the values in the corresponding scoring vector for each nucleotide position within its span. For example, a complete feature with coordinates *a *to *b *would be scored like so:

$$Score(a,b)=\sum_{a<=i<=b}ScoringVector[i]+\sum_{\begin{array}{c} evidence\_end5'=a\\ evidence\_end3'=b \end{array}}featureLength*weight(evidence)$$

As each gene prediction or spliced alignment is dismantled into its component parts, the parts contribute the weight of that evidence to the scoring scheme. For example, a single spliced protein alignment is dismantled into the protein alignment segments and intervening gaps, possibly contributing to feature types exon and intron of feature class PROTEIN. Those 'perfect' complete introns and exons yielded by dismantling of this protein alignment chain are added to the candidate exon and intron feature set if those features do not already exist. Each protein alignment segment contributes its corresponding evidence weight to each overlapping nucleotide position in the exon feature type scoring vector. Those protein alignment gaps that correspond to complete introns in our feature set contribute a value of (weight × length) to the feature-specific score of each corresponding intron.

The abundance of evidence is reflected in both the feature-specific and vectored scores. For example, often many protein homologies will exist at a given locus. Each protein database match (accession) at a given locus is scored separately, and so exon and introns supported by vast quantities of evidence will have scores that reflect both the weight and abundance of that evidence.

For the purpose of scoring exons and introns and minimizing the memory requirements required for storing the scoring vectors, each strand and associated set of evidence is initially examined separately; note that our final gene prediction examines both strands simultaneously. During the initial strand-based analysis, distinct exons and introns are collected from the evidence restricted to the strand being analyzed and scored accordingly. After collecting properly scored gene structure components from each strand, they are grouped together as a single collection of features from both DNA strands.

Dynamic programming is used to find the highest scoring set of connected exons, introns, and intergenic regions across the entire genome sequence (see Figure 5). Unlike exon and intron features, the intergenic features are not precomputed and are instead scored during the dynamic programming stage; scores for intergenic regions are computed when attempting to connect candidate gene termini while building the directed acyclic graph of connectable feature components (also referred to as the feature trellis). The highest scoring path of connected features is extracted from the feature trellis and separated into the individual gene predictions. A primary restriction within our feature trellis is that the introns connecting exons must exist as explicit components of our feature set; EVM will not connect two otherwise compatible exons unless the required intron exists within the inputted evidence, such as provided by a gene prediction, or spliced protein or transcript alignment.

Note that, by default, EVM will re-examine long introns to identify candidate nested genes. Although we find this functionality extraordinarily useful for automated annotation, especially for insect genomes, this function was not employed in any analysis described here. Although improvements in sensitivity can result from the nested gene search, there are associated costs in specificity (data not shown).

### Augmenting intergenic scores from approximate beginnings and ends of genes

Because the ABINITIO_PREDICTION class of evidence is the only class that contributes explicitly to the prediction of intergenic regions, coping with cases in which the consensus of *ab initio *predictions merges multiple adjacent genes into a single gene structure is particularly problematic. To split the merged consensus into separate individual predictions, the true intergenic region would need a score that is suitable to offset the alternative, typically involving a predicted intron that joins what should be distinct loci. To encourage the selection of separate complete gene structures supported by protein homologies instead of the merged gene, EVM augments the scores of intergenic regions supported indirectly by protein evidence, as elaborated below.

The approximate boundaries of candidate intergenic regions supported by protein homologies are localized by examining the boundaries of protein alignment chains. The beginnings and ends of all PROTEIN evidence structures (the far bounds of all spliced alignment chains, not the individual segments) are tallied. A sliding window of 300 nucleotides is applied to each strand, and all peaks of beginnings and ends are separately tallied. In addition to the protein alignment chains, the terminal exons provided by the extraction of long ORFs from PASA alignment assemblies also contribute to the tally of candidate beginnings and ends of genes.

From each begin peak, a corresponding initial exon is located from the feature set. The intergenic score for each nucleotide from the candidate initial exon upstream to the preceding gene is set to the maximal intergenic score, corresponding to the sum of the weights for ABINITIO_PREDICTION evidence classes. Likewise, from each candidate gene end, a terminal exon is located from the feature set, and the genome region downstream to the next gene is set to the maximal intergenic score. Note that single exon genes are also treated similarly as initial or terminal exons in the search for the next possible adjacent gene structure.

Although this search for gene boundaries is not very precise, the heuristic employed here tends to work acceptably well in practice. Choosing the proper boundaries of a gene structure is critical for predicting the entire gene correctly, as demonstrated by the greater variability in initial and terminal exon prediction among the various *ab initio *gene prediction programs.

### Filtering EVM predictions with low support

Instead of reporting the single best scoring gene structure at each locus, EVM reports the set of gene structures that, when connected together with the intervening intergenic regions, provides an optimal cumulative score. There are sometimes cases in which low scoring adventitious genes are included in the preliminary EVM gene set, largely as a consequence of ABINITIO_PREDICTION introns called on either strand in what are really intergenic regions. To remove these adventitious genes from the EVM gene set, the score of each EVM prediction is re-examined in the context of *ab initio *predicted introns being scored as if they were intergenic regions. An alternative noncoding score is computed for each EVM gene prediction by summing the predicted intergenic regions with the *ab initio *predicted intron regions. This noncoding score is then compared with the initial EVM prediction score, and those EVM predictions with a coding/noncoding score ratio below 0.75 are eliminated. An example of a low scoring EVM prediction removed during this postprocessing stage is illustrated in Additional data file 1 (Figure S5). An option is available in the EVM software to report these eliminated genes. In those cases in which all predictions agree, predictions lack introns, and the corresponding intergenic score is zero, the score ratio is set to an arbitrary high value and reported accordingly.

### Evaluating prediction accuracy

Gene prediction accuracy (sensitivity and specificity) was computed at the level of nucleotides, exons, transcripts, and complete genes, as described previously [10] but with slight modifications. Although some gene structures include untranslated region annotations, only the protein-coding portions of each exon were considered when we computed accuracy.

In our evaluation of the reference gene structures in rice, alternative splicing was ignored, and no attempt was made to generate a reference gene set for rice that included alternatively spliced transcripts. Therefore, given the one transcript per gene in the rice dataset, gene prediction accuracy calculations would necessarily be identical to the transcript accuracy calculations, and so only the gene prediction accuracy was reported. Although each reference gene region was provided as input to EVM in the context of the flanking 30 kb of genome sequence and corresponding evidence, all accuracy calculations were based on the gene predictions isolated from reference gene region including a flanking 500 bp. In our comparison of the accuracy of EVM to the annotation tools Glean and JIGSAW, we obtained the most current versions of the software available from their respective sites, namely version 3.2.9 for JIGSAW [55] and version 1.0.1 for GLEAN [56], downloaded directly from the subversion source repository.

Accuracy calculations on the human ENCODE genome regions included these regions and corresponding predictions in their entirety. Given that the GENCODE annotations included alternatively spliced transcripts, the prediction of alternatively spliced genes was a major component of our analysis, and so transcript prediction accuracy calculations were reported along with complete gene, exon, and nucleotide prediction accuracies.

### Estimating optimal evidence weights

The EVM training process is divided into three phases described below:

#### Initially optimized PREDICTION weights

In the first stage, optimal weights are explored for the ABINITIO_PREDICTION class in isolation from evidence of the other classes. The proper balance between the evidence weights applied to exons, introns, and intergenic regions is explored to optimize gene prediction accuracy. Weights are randomly chosen for each *ab initio *gene prediction type and normalized so that they sum to one. EVM is applied to each reference gene and specified length of flanking region included. EVM prediction accuracy is measured, and a conglomerate accuracy score is computed as follows:

AccuracyScore = F + gSn + eSn

Where F = (2 × nSn × nSp)/(nSn + nSp), Sn = TP/(TP + FN), and Sp = TP/(TP + FP). (TP, FP, FN correspond to true positives, false positives, and false negatives, respectively. The nSn and nSp indicate nucleotide sensitivity and specificity, respectively.)

Twenty random trials are performed. The weight combination that yielded the greatest AccuracyScore is chosen. These weight values are gradually adjusted while applying gradient ascent to find weight values that improve performance.

#### Initially optimized best individual evidence weights

Using the combination of weights now temporarily fixed for the ABINITIO_PREDICTION evidence, each other evidence type is introduced separately to find the minimum corresponding weight that provides the greatest AccuracyScore in the context of the ABINITIO_PREDICTION types. The weight for the other evidence type is first set to zero and evaluated. Next, the weight is set to the average weight value of the ABINITIO_PREDICTION types and evaluated. Gradient ascent is performed to explore adjusted weight values and a higher scoring weight. The minimum weight value that yielded the highest AccuracyScore is initially assigned to the other evidence type.

#### Simultaneous application of all evidence and relative weight refinements

The weight values for all evidence types are adjusted to find weight combinations that demonstrate improved prediction accuracies when all evidence is examined simultaneously. Evidence types are examined in descending order of their initially set weight values computed from phase 1 (ABINITIO_PREDICTION) or phase 2 (other) above. Weight values are gradually adjusted and gradient ascent is applied to explore better performing weight value in the context of the other evidence types. Cycling through the evidence types in this manner occurs until no appreciable improvement in performance is observed, in which case the training process ceases and the final weight values are reported.

Evidence weights and EVM prediction accuracies encountered during the training process using the rice data are illustrated in Additional data file 1 (Figure S6).

### Manual annotation of gene structures

The genome sequence, *ab initio *gene predictions, protein alignments, GeneWise predictions, and other plant EST alignments were examined using the Neomorphic/Affymetrix Annotation Station software (described by Haas and coworkers [28]). No rice transcript alignments either alone or in the context of PASA assemblies were made available to users so that we could reasonably estimate optimal gene structure annotation accuracy in the context of *ab initio *gene predictions and homologies to sequences derived from other organisms. A group of annotators were provided with the same data sets evaluated by EVM, only in graphical form. Annotators were instructed to model a gene structure in the targeted region that best reflected the available evidence using the Annotation Station software. Annotators were not allowed to examine the data deeper than the visual display provided. The sequence alignments themselves were not available except in the context of the glyphs highlighting their end points, and no additional sequence analyses such as running blast was allowed. The focus of this effort was not to measure the maximal accuracy of manual gene annotation accuracy in general, but only to measure the maximal possible accuracy of an automated annotation such as EVM given the restricted inputs.

## Abbreviations

bp = base pairs; EGASP = ENCODE Genome Annotation Assessment Project; ENCODE = ENCyclopedia of DNA Elements; eSn = exon sensitivity; eSp = exon specificity; EST = expressed sequence tag; EVM = EVidenceModeler; FL-cDNA = full-length cDNA; FN = false negatives; gSn = gene sensitivity; gSp = gene specificity; HMM = hidden Markov model; kb = kilobase; nSn = nucleotide sensitivity; nSp = nucleotide specificity; ORF = open reading frame; PASA = Program to Assemble Spliced Alignments; TN = true negatives; TP = true positives.

## Authors' contributions

BJH carried out all analyses, software development, and wrote the initial version of the manuscript while under the guidance of JW, OW, and SLS. SLS, MP, and JA helped to develop many of the underlying concepts of EVM. Analyses using the rice genome data were assisted by WZ and CRB. JO was responsible for generating all evidence for the rice and human genome sequences. All authors contributed to and approved the final version of the manuscript.

## Additional data files

The following additional data are available with the online version of this paper. Additional data file 1 includes the supplementary figures described throughout the report. Additional data file 2 contains supplementary data tables.

## Supplementary Material

Additional file 1 Supplementary figures. (Figure S1 shows the difference in rice gene prediction accuracy between using trained and intuitively set evidence weights. Figure S2 shows the change in human gene prediction accuracy due to application of PASA. Figure S3 shows the comparison of 1,058 reference gene structure exon distribution to all rice gene annotations. Figure S4 shows the gene prediction accuracies for EGASP gene sets. Figure S5 shows filtering EVM predictions with low support. Figure S6 shows optimization of evidence weights by exploring weight and evidence combinations. Click here for file

Additional file 2 Supplementary data tables. Table S1 provides trained weights for evidence based on evaluating 500 rice gene structures. Table S2 shows the gene prediction accuracy for EVM measured using 500 reference rice gene structures. Table S3 provides trained EVM weights including PASA. Table S4 provides trained EVM evidence weights for the ENCODE regions. Table S5 shows the EVM prediction accuracy using trained evidence weights for ENCODE regions. Click here for file
