# Supplementary material for: Automated eukaryotic gene structure annotation using EVidenceModeler and the Program to Assemble Spliced Alignments
Source: Genome Biol. 2008 Jan 11;9(1):R7. doi: 10.1186/gb-2008-9-1-r7 (PMC2395244; doi:10.1186/gb-2008-9-1-r7)
Supplement: Additional file 1 — Supplementary figures. (Figure S1 shows the difference in rice gene prediction accuracy between using trained and intuitively set evidence weights. Figure S2 shows the change in human gene prediction accuracy due to application of PASA. Figure S3 shows the comparison of 1,058 reference gene structure exon distribution to all rice gene annotations. Figure S4 shows the gene prediction accuracies for EGASP gene sets. Figure S5 shows filtering EVM predictions with low support. Figure S6 shows optimization of evidence weights by exploring weight and evidence combinations. [file gb-2008-9-1-r7-S1.pdf]

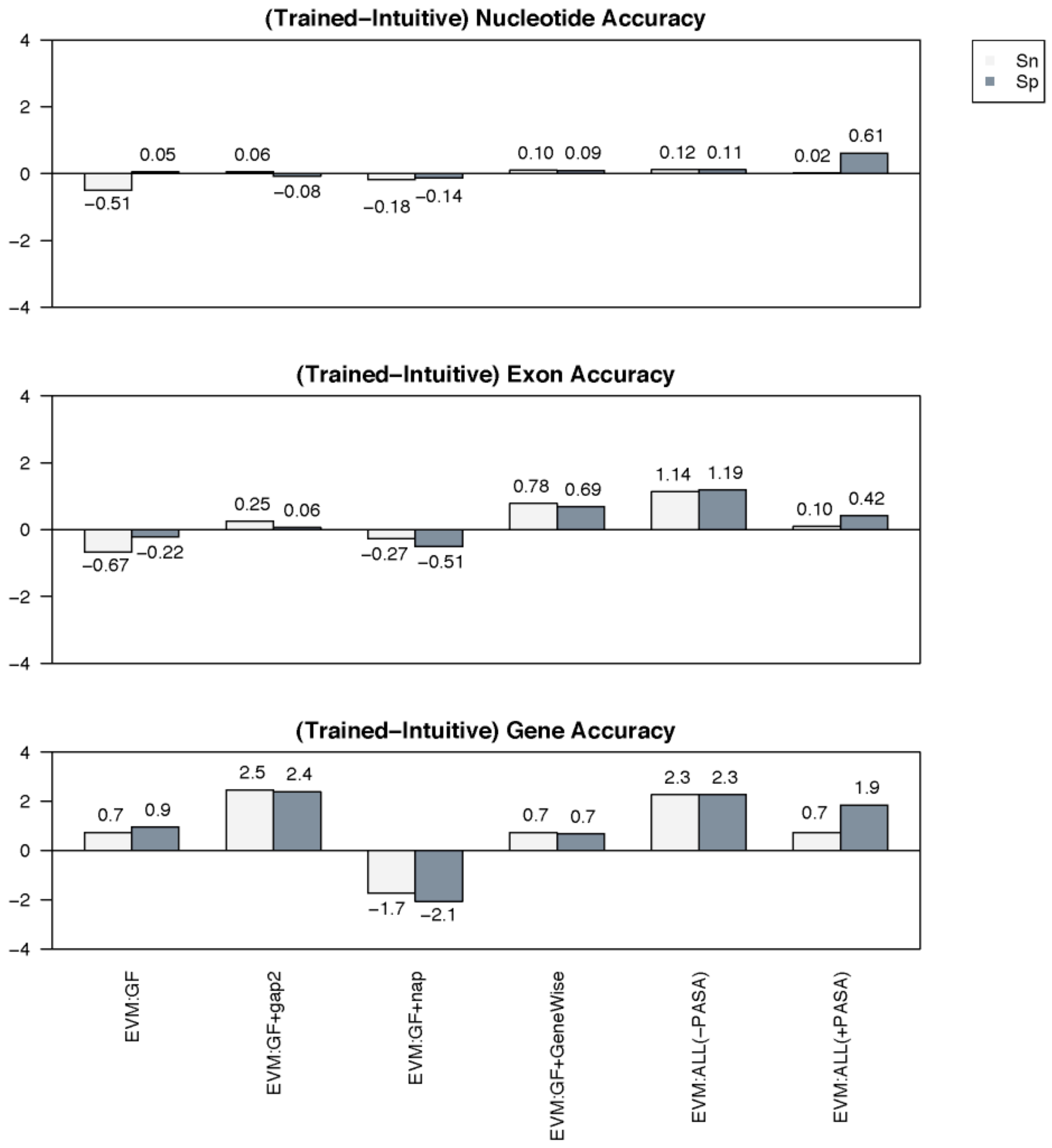

Figure S1: Difference in Rice Gene Prediction Accuracy Between Using Trained and Intuitively Set Evidence Weights. The difference in prediction accuracy between using trained weights versus using the intuitively set weights with EVM are marginal, with less than a three percent difference in each case.

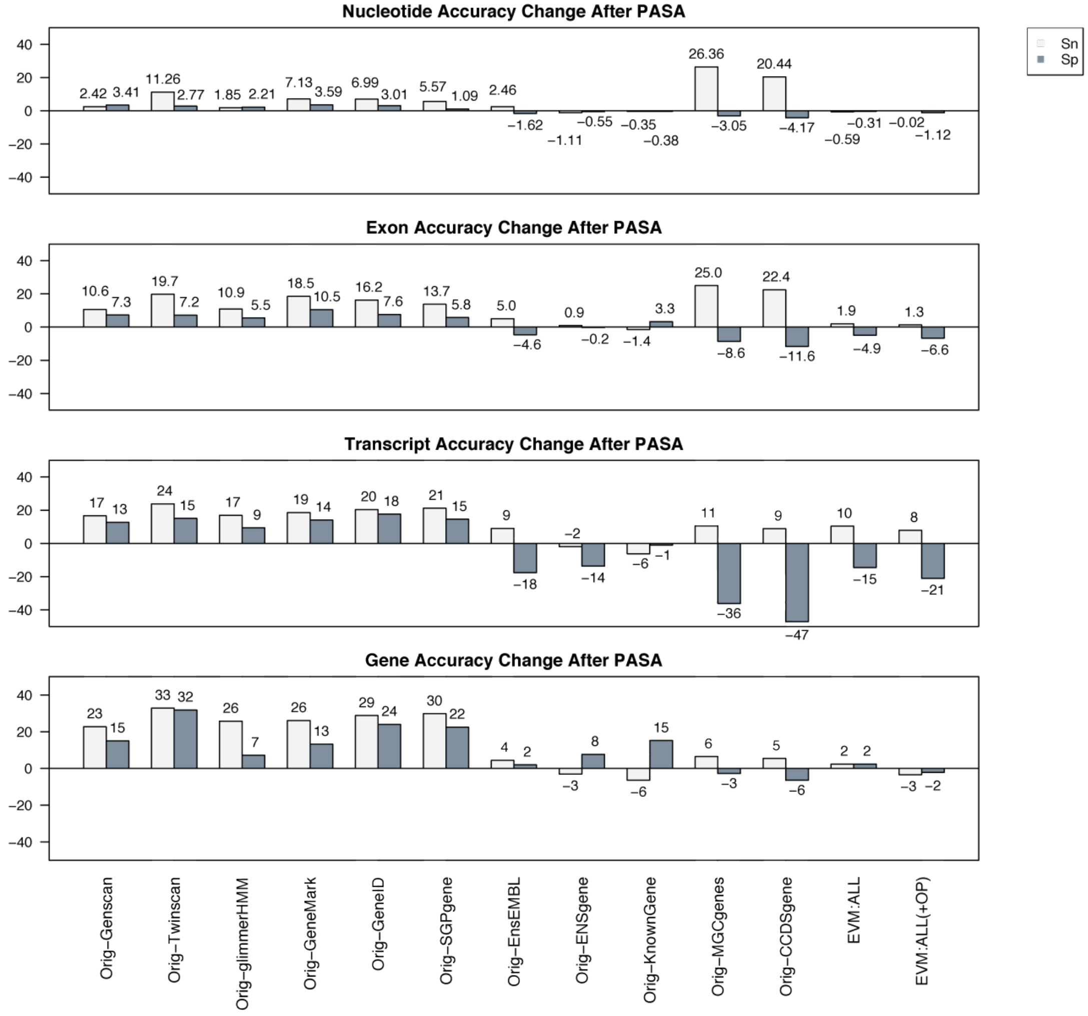

Figure S2: Change in Human Gene Prediction Accuracy Due to Application of PASA. The change in gene prediction accuracy resulting from an application of PASA is shown. PASA improves gene prediction accuracy when applied to the *ab initio* gene predictions. Results vary in the context of homology predictions, typically involving an increase in tSn and a greater decrease in tSp, reflecting differences in the annotation of alternative splicing isoforms between PASA and the human GENCODE annotations.

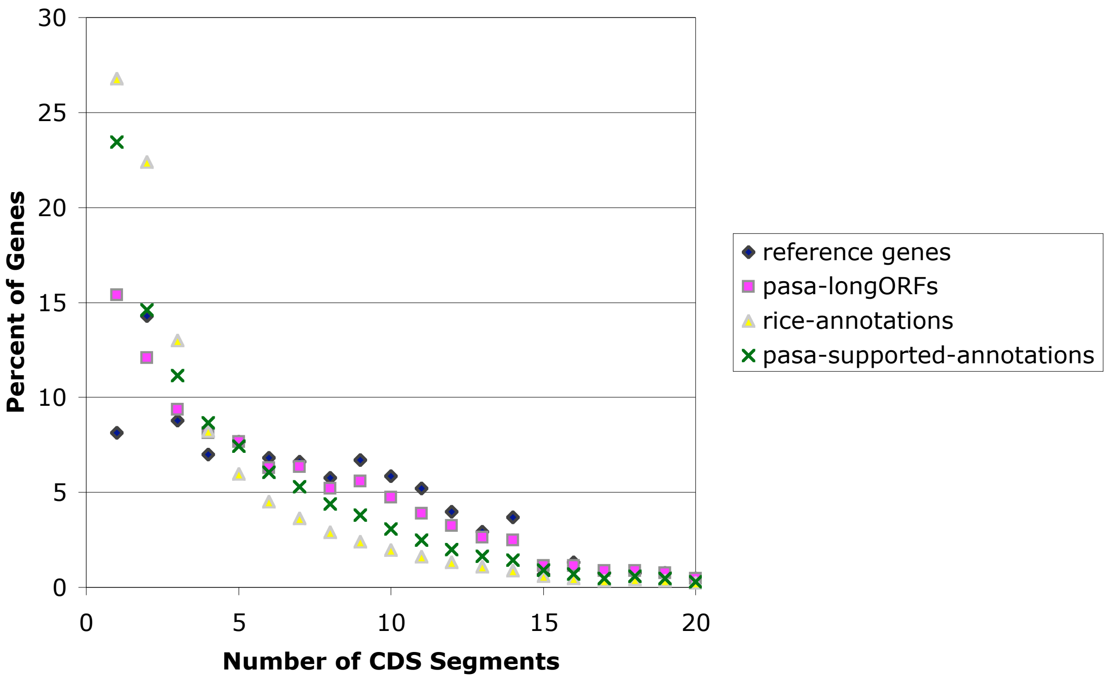

Figure S3: Comparison of the 1058 Reference Gene Structure Exon Distribution to All Rice Gene Annotations. The protein-coding exon (CDS segment) distribution is shown for the cDNA-supported manually confirmed 1058 reference rice gene structures, all existing rice gene structure annotations, the cDNA-supported (PASA-supported) subset of the rice gene structure annotations, and long ORFs found within PASA alignment assemblies. The reference gene structures have a similar CDS segment count distribution to the alternate sets of rice gene structures, albeit notably deficient in single-exon genes.

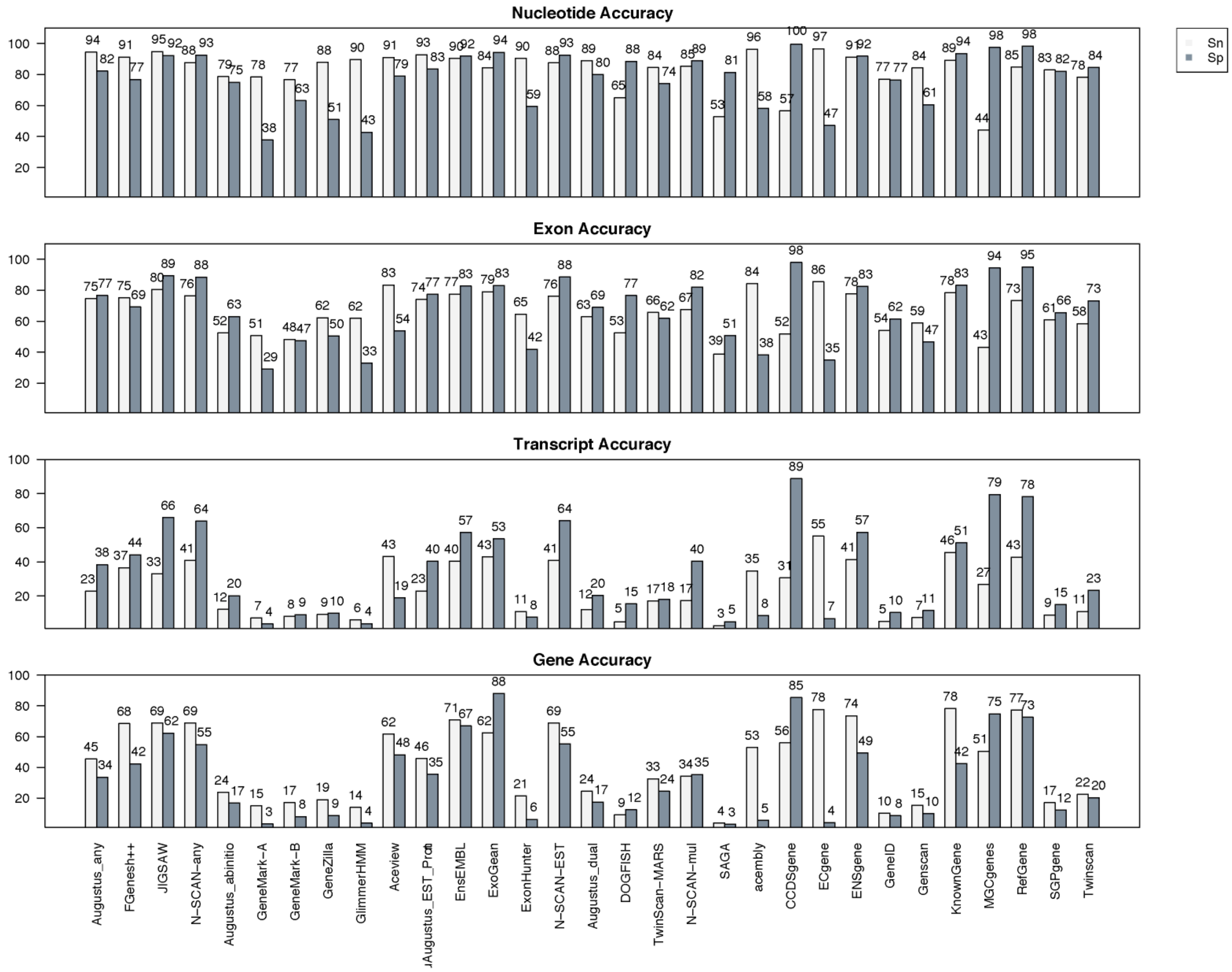

Figure S4: Gene Prediction Accuracies for EGASP Gene Sets. Gene prediction accuracies for each of the EGASP data sets were recomputed using reformatted data files and newly developed accuracy evaluation tools provided in the EVM software distribution. Recomputed accuracy values are mostly consistent with those reported earlier[10].

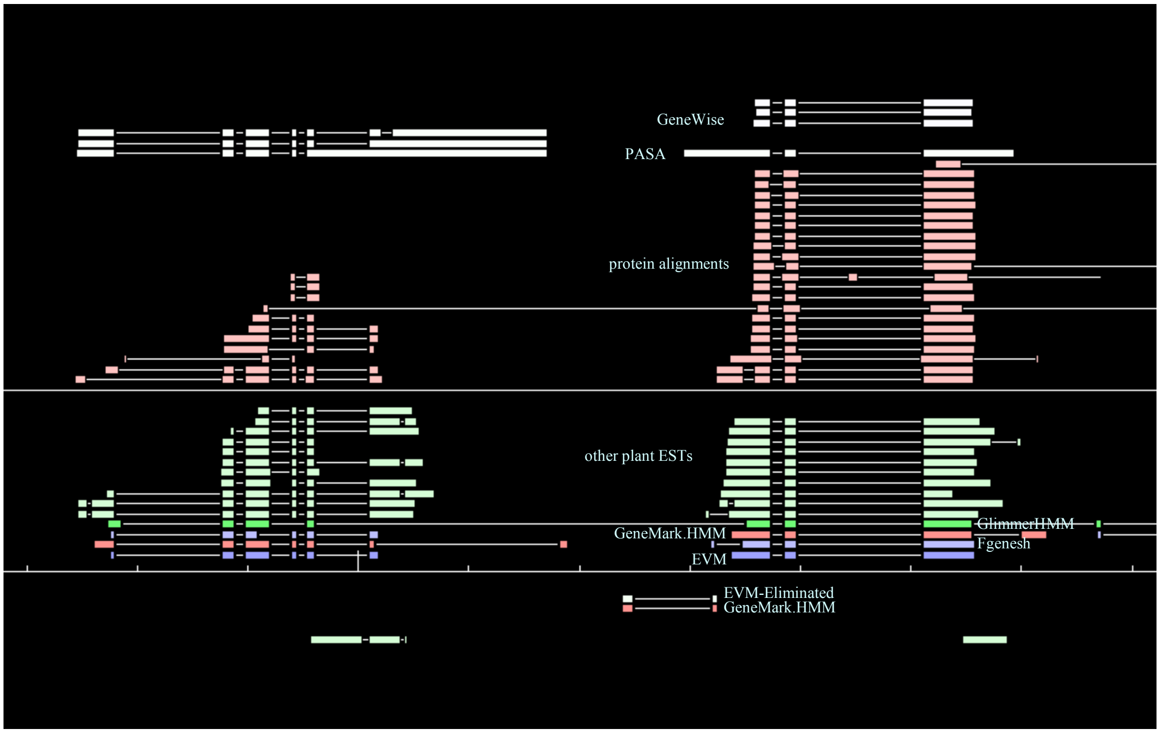

Figure S5: Filtering EVM Predictions With Low Support. The EVM prediction located centrally and on the reverse strand was based on a two-exon GeneMark.hmm prediction. It was initially selected as part of the highest scoring parse of connected exon structures because there was little evidence support for the alternative: Fgenesh encodes an overlapping coding exon on the opposite strand and predicts the remainder of the EVM-predicted region as intergenic; GlimmerHMM predicts an intron across the entire predicted region from the opposite strand. During EVMs post-processing stage where consensus predictions are rescored with introns and intergenic regions scored cumulatively as noncoding, the noncoding score strongly outweighs the coding score for this prediction, and so it is eliminated from the EVM prediction set. The image shown is a screenshot of rice contig #101 as viewed using the *TkGFF3_viewer.pl* software included in the EVM software distribution.

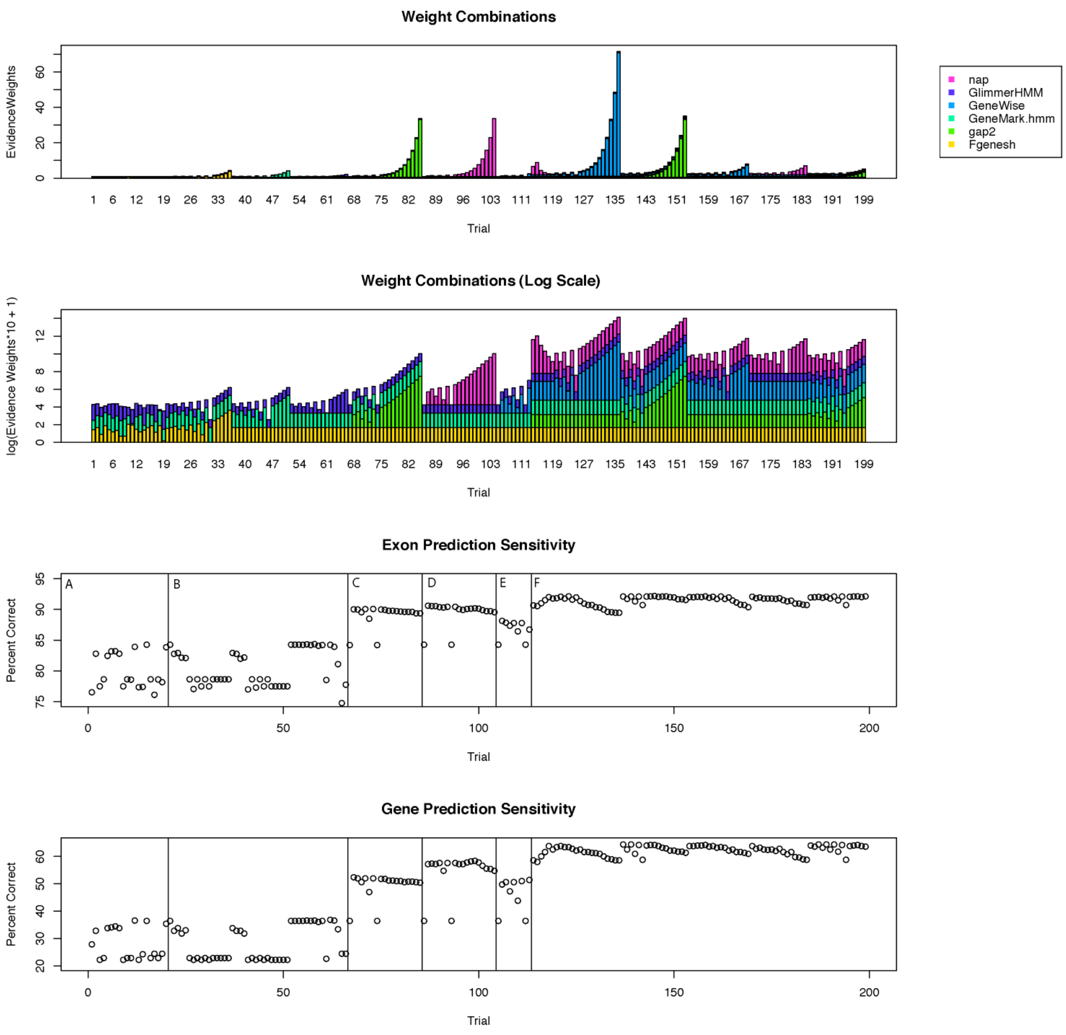

Figure S6: Optimization of Evidence Weights by Exploring Weight and Evidence Combinations. The weight combinations explored during an episode of weight training using 500 rice gene structures is shown. Each data point provides the average gene prediction accuracy resulting from an invocation of EVM on the 500 reference gene structures used for training. The three phases of training are shown by the partitions A-F. Phase I involves partition A in which random weight combinations are explored for the *ab initio* predictions, and partition B, where gradient ascent is performed using the best scoring combination from partition A. Phase II (partitions C-E) involves separately introducing each of the other evidence types with the *ab initio* predictions and best scoring weight combination from partition B. Partition C introduces gap2 alignments of

other plant gene indices, partition D introduces nap alignments of non-rice proteins, and partition E introduces genewise predictions based on non-rice proteins homologies. Phase III corresponds to the final partition E whereby all evidences are examined in concert with their weights initially set to their highest scoring setting from the earlier partitions, and gradient ascent is applied to find a more optimal combination of weight values.
