# Supplementary material for: Automated eukaryotic gene structure annotation using EVidenceModeler and the Program to Assemble Spliced Alignments
Source: Genome Biol. 2008 Jan 11;9(1):R7. doi: 10.1186/gb-2008-9-1-r7 (PMC2395244; doi:10.1186/gb-2008-9-1-r7)
Supplement: Additional file 2 — Supplementary data tables. Table S1 provides trained weights for evidence based on evaluating 500 rice gene structures. Table S2 shows the gene prediction accuracy for EVM measured using 500 reference rice gene structures. Table S3 provides trained EVM weights including PASA. Table S4 provides trained EVM evidence weights for the ENCODE regions. Table S5 shows the EVM prediction accuracy using trained evidence weights for ENCODE regions. [file gb-2008-9-1-r7-S2.pdf]

Trial 1				
	#ev_type	phase_1	phase_2	phase_3
	genemark	0.425582322		0.425582322
	fgenesh	0.428677512		0.428677512
	glimmerHMM	0.145740166		0.145740166
	gap2-plant_gene_index.11282006.fasta		0.333333333	0.333333333
	nap-nr_minus_rice.fasta		4.604817708	0.575602214
	genewise-nr_minus_rice.fasta		0.708333333	0.708333333
Trial 2				
	#ev_type	phase_1	phase_2	phase_3
	genemark	0.417757451		0.417757451
	fgenesh	0.428940503		0.428940503
	glimmerHMM	0.252288315		0.252288315
	gap2-plant_gene_index.11282006.fasta		0.366328757	0.366328757
	nap-nr_minus_rice.fasta		2.452685504	0.613171376
	genewise-nr_minus_rice.fasta		0.641075324	0.641075324
Trial 3				
	#ev_type	phase_1	phase_2	phase_3
	genemark	0.369787882		0.369787882
	fgenesh	0.401023028		0.401023028
	glimmerHMM	0.387329562		0.387329562
	gap2-plant_gene_index.11282006.fasta		0.386046824	0.579070236
	nap-nr_minus_rice.fasta		0.193023412	0.681613924
	genewise-nr_minus_rice.fasta		5.333025754	0.33331411

Table S1: Trained Weights for Evidence Based on Evaluating 500 Rice Gene Structures

Trial 1								
	#ev_type	gene count	Nucleotide Sensitivity	Nucleotide Specificity	Exon Sensitivity	Exon Specificity	Transcript Sensitivity	Transcript Specificity
	just_genefinders	565	94.87	96.46	83.43	81.42	38.4	33.98
	gap2-plant_gene_index	551	96.91	98.18	90.3	87.38	54	49
	nap-nr_minus_rice	576	98.23	97.52	89.17	86.97	55.8	48.44
	genewise-nr_minus_rice	559	96.95	97.07	86.67	85.53	48	42.93
	final_weights_minus_pasa	555	98.4	98.02	91.98	90.25	61.8	55.68
	final_weights_plus_pasa	540	99.94	99.47	99.65	98.29	97.8	90.56
Trial 2								
	#ev_type	gene count	Nucleotide Sensitivity	Nucleotide Specificity	Exon Sensitivity	Exon Specificity	Transcript Sensitivity	Transcript Specificity
	just_genefinders	560	94.88	96.55	83.46	81.68	37.8	33.75
	gap2-plant_gene_index	547	96.94	98.27	90.3	87.61	53.6	48.99
	nap-nr_minus_rice	567	98.25	97.71	89.51	87.56	56.8	50.09
	genewise-nr_minus_rice	554	96.86	97.18	86.96	86.14	49.2	44.4
	final_weights_minus_pasa	554	98.41	98.04	92	90.33	62	55.96
	final_weights_plus_pasa	538	99.95	99.48	99.68	98.37	98	91.08
Trial 3								
	#ev_type	gene count	Nucleotide Sensitivity	Nucleotide Specificity	Exon Sensitivity	Exon Specificity	Transcript Sensitivity	Transcript Specificity
	just_genefinders	561	94.93	96.66	83.92	82.14	37.6	33.51
	gap2-plant_gene_index	544	96.63	98.28	90.24	87.85	53.4	49.08
	nap-nr_minus_rice	555	97.57	97.82	90.18	88.64	55.6	50.09
	genewise-nr_minus_rice	557	96.79	97.12	86.21	85.66	49	43.99
	final_weights_minus_pasa	552	98.54	98.13	92.58	90.85	63.6	57.61
	final_weights_plus_pasa	536	99.95	99.52	99.68	98.45	98	91.42
Averages								
	#ev_type	gene count	Nucleotide Sensitivity	Nucleotide Specificity	Exon Sensitivity	Exon Specificity	Transcript Sensitivity	Transcript Specificity
	just_genefinders	562	94.89333333	96.55666667	83.60333333	81.74666667	37.93333333	33.74666667
	gap2-plant_gene_index	547.3333333	96.82666667	98.24333333	90.28	87.61333333	53.66666667	49.02333333
	nap-nr_minus_rice	566	98.01666667	97.68333333	89.62	87.72333333	56.06666667	49.54
	genewise-nr_minus_rice	556.6666667	96.86666667	97.12333333	86.61333333	85.77666667	48.73333333	43.77333333
	final_weights_minus_pasa	553.6666667	98.45	98.06333333	92.18666667	90.47666667	62.46666667	56.41666667
	final_weights_plus_pasa	538	99.94666667	99.49	99.67	98.37	97.93333333	91.02
StDevs								
	#ev_type	gene count	Nucleotide Sensitivity	Nucleotide Specificity	Exon Sensitivity	Exon Specificity	Transcript Sensitivity	Transcript Specificity
	just_genefinders	1	0.025891512	0.061674174	0.235379914	0.248581159	0.167774099	0.137611907
	gap2-plant_gene_index	1.835856849	0.156855678	0.018954136	0.030550505	0.137611907	0.138777733	0.045501323
	nap-nr_minus_rice	6.658328118	0.345532332	0.072444104	0.359304884	0.582144821	0.604918115	0.317542648
	genewise-nr_minus_rice	1.644294287	0.042470033	0.03371998	0.375356621	0.250340509	0.234125639	0.318264972
	final_weights_minus_pasa	1.071516751	0.066583281	0.046706332	0.296073064	0.26810722	0.822822604	0.851967222
	final_weights_plus_pasa	1.154700538	0.0019245	0.02081666	0.005773503	0.046188022	0.038490018	0.215715862

Table S2: Gene Prediction Accuracy for EVM Measured Using 500 Reference Rice Gene Structures.

	Fgenesh	GeneMark.hmm	GlimmerHMM	gap2	nap	GeneWise	PASA
EVM-GF	0.391	0.371	0.352	NA	NA	NA	NA
EVM-gap2	0.391	0.371	0.352	0.371	NA	NA	NA
EVM-nap	0.391	0.371	0.352	NA	0.650	NA	NA
EVM-genwise	0.391	0.371	0.352	NA	NA	5.13	NA
EVM-all	0.391	0.371	0.352	0.371	0.650	5.13	NA
EVM-all+PASA	0.391	0.371	0.352	0.371	0.650	5.13	15.31

Table S3: Trained EVM Weights Including PASA. The above weights were used to evaluate EVM and compare EVM's accuracy to the alternative annotation tools Glean and JIGSAW.

Trial 1			Phase I	Phase II	Phase III(-OP)	Phase III (+OP)
	ABINITIO_PREDICTION	Twinscan	0.254839987		0.218631568	0.218631568
	ABINITIO_PREDICTION	GenemarkMasked	0.241934531		0.207559757	0.207559757
	ABINITIO_PREDICTION	glimmerHMM	0.216024881		0.185331427	0.185331427
	ABINITIO_PREDICTION	Genscan	0.195926394		0.168088592	0.168088592
	ABINITIO_PREDICTION	SGP	0.136837472		0.117395199	0.117395199
	ABINITIO_PREDICTION	GeneID	0.120050604		0.102993459	0.102993459
	TRANSCRIPT	gap2-DOGGI.091906.fasta		7.526645402	2.025880159	4.051760317
	OTHER_PREDICTION	CDDSgene		4.286930652		2.212845078
	OTHER_PREDICTION	KnownGene		4.048767838		6.584750806
	OTHER_PREDICTION	MGCgenes		2.857953768		86.20486566
	TRANSCRIPT	gap2-MGI.091806.fasta		2.409647295	0.327828901	0.655657801
	OTHER_PREDICTION	ENSgene		2.01737913		2.212845078
	OTHER_PREDICTION	EnsEMBL		2.01737913		2.212845078
	TRANSCRIPT	gap2-RGI.091806.fasta		1.046048438	1.147401152	1.147401152
	TRANSCRIPT	alignAssembly-egasp_02232007_pasa		0.896612947	1.475230052	0.491743351
	TRANSCRIPT	gap2-BTGI.091806.fasta		0.896612947	0.655657801	4.389833871
	TRANSCRIPT	gap2-CINGI.100404.fasta		0.625415204	0.686012329	0.686012329
	TRANSCRIPT	gap2-XGI.100604.fasta		0.597741965	0.783146818	0.783146818
	TRANSCRIPT	gap2-SSGI.092006.fasta		0.597741965	1.147401152	1.147401152
	PROTEIN	nap-nr.minus_human.pep		0.475979713	0.412821578	0.412821578
	PROTEIN	genewise-nr.minus_human.pep		0.265663095	0.437105201	0.291403467
	TRANSCRIPT	gap2-GGGI.092006.fasta		0.265663095	0.291403467	0.291403467
Trial 2						
	ABINITIO_PREDICTION	glimmerHMM	0.264888814		0.249271369	0.249271369
	ABINITIO_PREDICTION	GenemarkMasked	0.20327077		0.191286232	0.191286232
	ABINITIO_PREDICTION	Genscan	0.185459445		0.174525036	0.174525036
	ABINITIO_PREDICTION	Twinscan	0.172607799		0.162431104	0.162431104
	ABINITIO_PREDICTION	SGP	0.127450462		0.119936175	0.119936175
	ABINITIO_PREDICTION	GeneID	0.108975091		0.102550084	0.102550084
	TRANSCRIPT	gap2-DOGGI.091906.fasta		7.526645402	7.526645402	7.526645402
	OTHER_PREDICTION	CDDSgene		4.286930652		192.1711383
	OTHER_PREDICTION	KnownGene		4.048767838		18.28271727
	OTHER_PREDICTION	MGCgenes		2.857953768		2.857953768
	TRANSCRIPT	gap2-MGI.091806.fasta		2.409647295	3.614470942	2.409647295
	OTHER_PREDICTION	ENSgene		2.01737913		2.01737913
	OTHER_PREDICTION	EnsEMBL		2.01737913		2.01737913
	TRANSCRIPT	gap2-RGI.091806.fasta		1.046048438	1.046048438	1.046048438
	TRANSCRIPT	alignAssembly-egasp_02232007_pasa		0.896612947	0.896612947	1.905302512
	TRANSCRIPT	gap2-BTGI.091806.fasta		0.896612947	0.896612947	0.896612947
	TRANSCRIPT	gap2-CINGI.100404.fasta		0.625415204	0.625415204	0.625415204
	TRANSCRIPT	gap2-XGI.100604.fasta		0.597741965	0.597741965	0.597741965
	TRANSCRIPT	gap2-SSGI.092006.fasta		0.597741965	0.597741965	0.597741965
	PROTEIN	nap-		0.475979713	0.713969569	0.475979713

		nr.minus_human.pep				
	PROTEIN	genewise-nr.minus_human.pep		0.265663095	0.132831548	0.265663095
	TRANSCRIPT	gap2-GGGI.092006.fasta		0.265663095	0.265663095	0.265663095

Table S4: Trained EVM Evidence Weights for the ENCODE Regions

Egasp_Trial1		gene count	tpg	Nuc_Sn	Nuc_Sp	Exon_Sn
	./CDDSgene.weights.NoRec.out.gff3.GTF.myEval	339	1	90.27	87.59	74.67
	./ENSgene.weights.NoRec.out.gff3.GTF.myEval	348	1	91.45	87.13	77.31
	./EnsEMBL.weights.NoRec.out.gff3.GTF.myEval	349	1	91.61	86.98	77.53
	./KnownGene.weights.NoRec.out.gff3.GTF.myEval	358	1	93.17	87.1	79.44
	./MGCgenes.weights.NoRec.out.gff3.GTF.myEval	342	1	89.01	87.63	72.9
	./alignAssembly.weights.NoRec.out.gff3.GTF.myEval	373	1	92.39	85.75	79.05
	./final_all.weights.NoRec.out.gff3.GTF.myEval	402	1	94.05	83.42	81.72
	./final_minus_homologypreds.weights.NoRec.out.gff3.GTF.myEval	400	1	94.76	82.75	80.6
	./gap2-BTGI.weights.NoRec.out.gff3.GTF.myEval	322	1	86.18	86.92	69.65
	./gap2-CINGI.weights.NoRec.out.gff3.GTF.myEval	323	1	81.81	85.11	61.45
	./gap2-DOGGI.weights.NoRec.out.gff3.GTF.myEval	321	1	83.08	87	65.68
	./gap2-GGGI.weights.NoRec.out.gff3.GTF.myEval	323	1	83.2	85.54	64.38
	./gap2-MGI.weights.NoRec.out.gff3.GTF.myEval	319	1	86.92	86.68	71.57
	./gap2-RGI.weights.NoRec.out.gff3.GTF.myEval	320	1	85.58	86.73	69.11
	./gap2-SSGI.weights.NoRec.out.gff3.GTF.myEval	322	1	84.86	88.32	67.7
	./gap2-XGI.weights.NoRec.out.gff3.GTF.myEval	326	1	82.36	85.06	62.39
	./genewise-nr.minus_human.pep.weights.NoRec.out.gff3.GTF.myEval	354	1	90.83	83.27	71.06
	./just_genefinders.weights.NoRec.out.gff3.GTF.myEval	323	1	81.46	85.01	60.87
	./nap-nr.minus_human.pep.weights.NoRec.out.gff3.GTF.myEval	371	1	94.89	84.63	78.11
Egasp_Trial2						
	./CDDSgene.weights.NoRec.out.gff3.GTF.myEval	351	1	90.79	86.26	75.29
	./ENSgene.weights.NoRec.out.gff3.GTF.myEval	355	1	91.64	86.28	77.42
	./EnsEMBL.weights.NoRec.out.gff3.GTF.myEval	356	1	91.8	86.15	77.64
	./KnownGene.weights.NoRec.out.gff3.GTF.myEval	370	1	92.65	86.26	79.3
	./MGCgenes.weights.NoRec.out.gff3.GTF.myEval	354	1	89.54	85.74	73.19
	./alignAssembly.weights.NoRec.out.gff3.GTF.myEval	384	1	92.63	83.93	79.19
	./final_all.weights.NoRec.out.gff3.GTF.myEval	433	1	94.11	82.05	81.65
	./final_minus_homologypreds.weights.NoRec.out.gff3.GTF.myEval	402	1	92.58	81.17	78.43
	./gap2-BTGI.weights.NoRec.out.gff3.GTF.myEval	338	1	87.81	85.13	70.85
	./gap2-CINGI.weights.NoRec.out.gff3.GTF.myEval	346	1	83.99	82.32	63.66
	./gap2-DOGGI.weights.NoRec.out.gff3.GTF.myEval	337	1	84.74	84.77	67.3
	./gap2-GGGI.weights.NoRec.out.gff3.GTF.myEval	344	1	85.29	82.96	66
	./gap2-MGI.weights.NoRec.out.gff3.GTF.myEval	338	1	89.61	84.77	73.84
	./gap2-RGI.weights.NoRec.out.gff3.GTF.myEval	336	1	87.67	84.81	71.1
	./gap2-SSGI.weights.NoRec.out.gff3.GTF.myEval	346	1	86.6	83.99	69.11
	./gap2-XGI.weights.NoRec.out.gff3.GTF.myEval	347	1	84.18	82.28	64.2
	./genewise-nr.minus_human.weights.NoRec.out.gff3.GTF.myEval	365	1	91.19	82.24	71.35
	./just_genefinders.weights.NoRec.out.gff3.GTF.myEval	346	1	83.66	82.16	63.15
	./nap-nr.minus_human.weights.NoRec.out.gff3.GTF.myEval	381	1	95.18	83.69	78.29
Average						
	./CDDSgene.weights.NoRec.out.gff3.GTF.myEval	345	1	90.53	86.925	74.98
	./ENSgene.weights.NoRec.out.gff3.GTF.myEval	351.5	1	91.545	86.705	77.365
	./EnsEMBL.weights.NoRec.out.gff3.GTF.myEval	352.5	1	91.705	86.565	77.585
	./KnownGene.weights.NoRec.out.gff3.GTF.myEval	364	1	92.91	86.68	79.37
	./MGCgenes.weights.NoRec.out.gff3.GTF.myEval	348	1	89.275	86.685	73.045
	./alignAssembly.weights.NoRec.out.gff3.GTF.myEval	378.5	1	92.51	84.84	79.12
	./final_all.weights.NoRec.out.gff3.GTF.myEval	417.5	1	94.08	82.735	81.685
	./final_minus_homologypreds.weights.NoRec.out.gff3.GTF.myEval	401	1	93.67	81.96	79.515
	./gap2-BTGI.weights.NoRec.out.gff3.GTF.myEval	330	1	86.995	86.025	70.25
	./gap2-CINGI.weights.NoRec.out.gff3.GTF.myEval	334.5	1	82.9	83.715	62.555
	./gap2-DOGGI.weights.NoRec.out.gff3.GTF.myEval	329	1	83.91	85.885	66.49
	./gap2-GGGI.weights.NoRec.out.gff3.GTF.myEval	333.5	1	84.245	84.25	65.19
	./gap2-MGI.weights.NoRec.out.gff3.GTF.myEval	328.5	1	88.265	85.725	72.705
	./gap2-RGI.weights.NoRec.out.gff3.GTF.myEval	328	1	86.625	85.77	70.105
	./gap2-SSGI.weights.NoRec.out.gff3.GTF.myEval	334	1	85.73	86.155	68.405
	./gap2-XGI.weights.NoRec.out.gff3.GTF.myEval	336.5	1	83.27	83.67	63.295
	./genewise-nr.minus_human.weights.NoRec.out.gff3.GTF.myEval	359.5	1	91.01	82.755	71.205
	./just_genefinders.weights.NoRec.out.gff3.GTF.myEval	334.5	1	82.56	83.585	62.01
	./nap-nr.minus_human.weights.NoRec.out.gff3.GTF.myEval	376	1	95.035	84.16	78.2

StDev						
	./CDDSgene.weights.NoRec.out.gff3.GTF.myEval	8.485281374	0	0.367695526	0.940452019	0.438406204
	./ENSgene.weights.NoRec.out.gff3.GTF.myEval	4.949747468	0	0.134350288	0.601040764	0.077781746
	./EnsEMBL.weights.NoRec.out.gff3.GTF.myEval	4.949747468	0	0.134350288	0.586898628	0.077781746
	./KnownGene.weights.NoRec.out.gff3.GTF.myEval	8.485281374	0	0.367695526	0.593969696	0.098994949
	./MGCgenes.weights.NoRec.out.gff3.GTF.myEval	8.485281374	0	0.374766594	1.336431816	0.205060967
	./alignAssembly.weights.NoRec.out.gff3.GTF.myEval	7.778174593	0	0.169705627	1.286934342	0.098994949
	./final_all.weights.NoRec.out.gff3.GTF.myEval	21.92031022	0	0.042426407	0.96873629	0.049497475
	./final_minus_homologypreds.weights.NoRec.out.gff3.GTF.myEval	1.414213562	0	1.541492783	1.117228714	1.534421715
	./gap2-BTGI.weights.NoRec.out.gff3.GTF.myEval	11.3137085	0	1.152584053	1.265721138	0.848528137
	./gap2-CINGI.weights.NoRec.out.gff3.GTF.myEval	16.26345597	0	1.541492783	1.97282792	1.562705986
	./gap2-DOGGI.weights.NoRec.out.gff3.GTF.myEval	11.3137085	0	1.173797257	1.576848122	1.145512986
	./gap2-GGGI.weights.NoRec.out.gff3.GTF.myEval	14.8492424	0	1.477853173	1.824335495	1.145512986
	./gap2-MGI.weights.NoRec.out.gff3.GTF.myEval	13.43502884	0	1.902117241	1.350573952	1.605132393
	./gap2-RGI.weights.NoRec.out.gff3.GTF.myEval	11.3137085	0	1.477853173	1.35764502	1.407142495
	./gap2-SSGI.weights.NoRec.out.gff3.GTF.myEval	16.97056275	0	1.230365799	3.061772363	0.997020561
	./gap2-XGI.weights.NoRec.out.gff3.GTF.myEval	14.8492424	0	1.286934342	1.965756852	1.279863274
	./genewise-nr.minus_human.weights.NoRec.out.gff3.GTF.myEval	7.778174593	0	0.254558441	0.728319985	0.205060967
	./just_genefinders.weights.NoRec.out.gff3.GTF.myEval	16.26345597	0	1.555634919	2.015254326	1.612203461
	./nap-nr.minus_human.weights.NoRec.out.gff3.GTF.myEval	7.071067812	0	0.205060967	0.664680374	0.127279221

Table S5: EVM Prediction Accuracy Using Trained Evidence Weights for ENCODE Regions
